# Supplementary material for: Identification, Phylogenetic and Expression Analyses of the AAAP Gene Family in Liriodendron chinense Reveal Their Putative Functions in Response to Organ and Multiple Abiotic Stresses
Source: Int J Mol Sci. 2022 Apr 26;23(9):4765. doi: 10.3390/ijms23094765 (PMC9100865; doi:10.3390/ijms23094765)
Supplement: Supplementary file 1 [file ijms-23-04765-s001.zip › Figure S1.pdf]

a

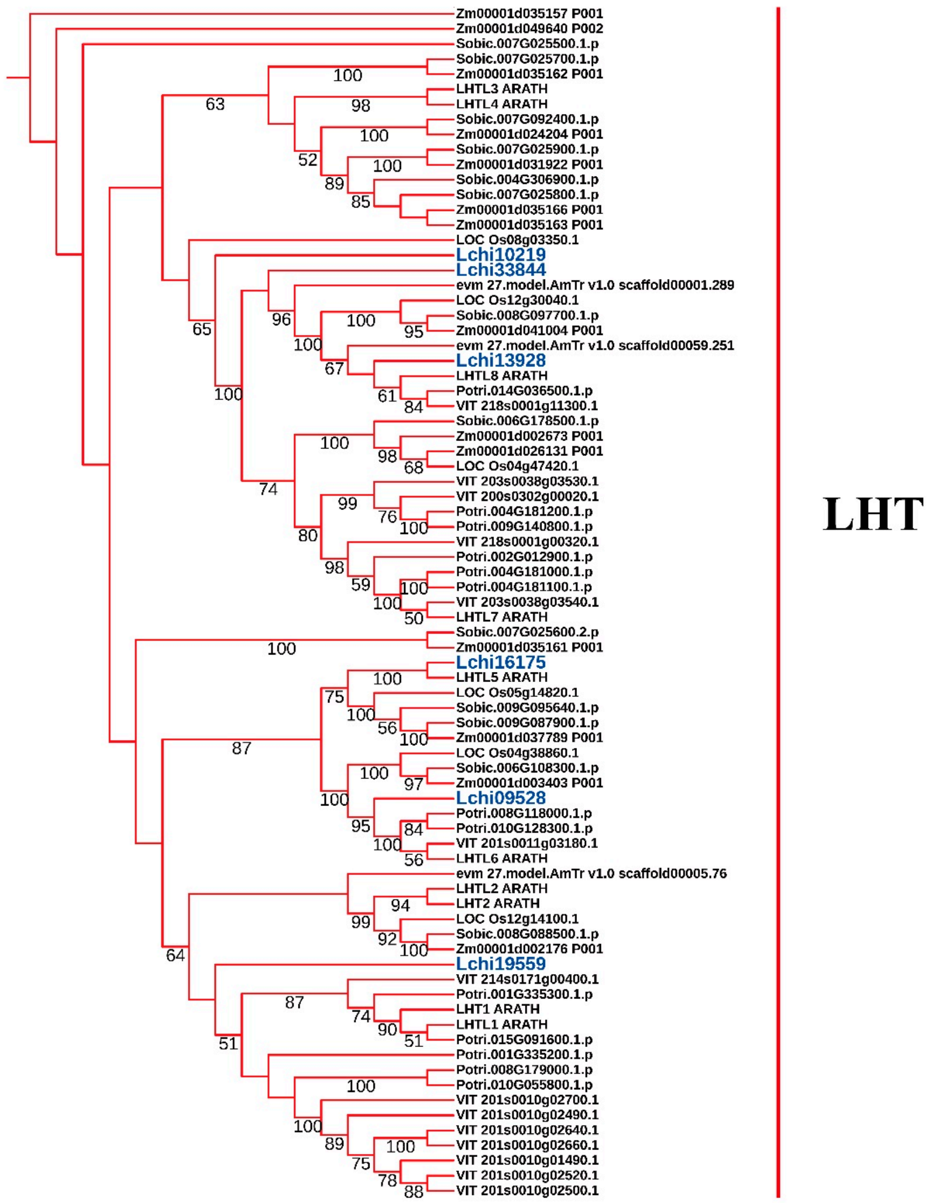

Zm00001d035157 P001
Zm00001d049640 P002
Sobic.007G025500.1.p
Sobic.007G025700.1.p
Zm00001d035162 P001
LHTL3 ARATH
LHTL4 ARATH
Sobic.007G092400.1.p
Zm00001d024204 P001
Sobic.007G025900.1.p
Zm00001d031922 P001
Sobic.004G306900.1.p
Sobic.007G025800.1.p
Zm00001d035166 P001
Zm00001d035163 P001
LOC Os08g03350.1
Lchi10219
Lchi33844
evm 27.model.AmTr v1.0 scaffold00001.289
LOC Os12g30040.1
Sobic.008G097700.1.p
Zm00001d041004 P001
evm 27.model.AmTr v1.0 scaffold00059.251
Lchi13928
LHTL8 ARATH
Potri.014G036500.1.p
VIT 218s0001g11300.1
Sobic.006G178500.1.p
Zm00001d002673 P001
Zm00001d026131 P001
LOC Os04g47420.1
VIT 203s0038g03530.1
VIT 200s0302g00020.1
Potri.004G181200.1.p
Potri.009G140800.1.p
VIT 218s0001g00320.1
Potri.002G012900.1.p
Potri.004G181000.1.p
Potri.004G181100.1.p
VIT 203s0038g03540.1
LHTL7 ARATH
Sobic.007G025600.2.p
Zm00001d035161 P001
Lchi16175
LHTL5 ARATH
LOC Os05g14820.1
Sobic.009G095640.1.p
Sobic.009G087900.1.p
Zm00001d037789 P001
LOC Os04g38860.1
Sobic.006G108300.1.p
Zm00001d003403 P001
Lchi09528
Potri.008G118000.1.p
Potri.010G128300.1.p
VIT 201s0011g03180.1
LHTL6 ARATH
evm 27.model.AmTr v1.0 scaffold00005.76
LHTL2 ARATH
LHT2 ARATH
LOC Os12g14100.1
Sobic.008G088500.1.p
Zm00001d002176 P001
Lchi19559
VIT 214s0171g00400.1
Potri.001G335300.1.p
LHT1 ARATH
LHTL1 ARATH
Potri.015G091600.1.p
Potri.001G335200.1.p
Potri.008G179000.1.p
Potri.010G055800.1.p
VIT 201s0010g02700.1
VIT 201s0010g02490.1
VIT 201s0010g02640.1
VIT 201s0010g02660.1
VIT 201s0010g01490.1
VIT 201s0010g02520.1
VIT 201s0010g02500.1
LHT

b

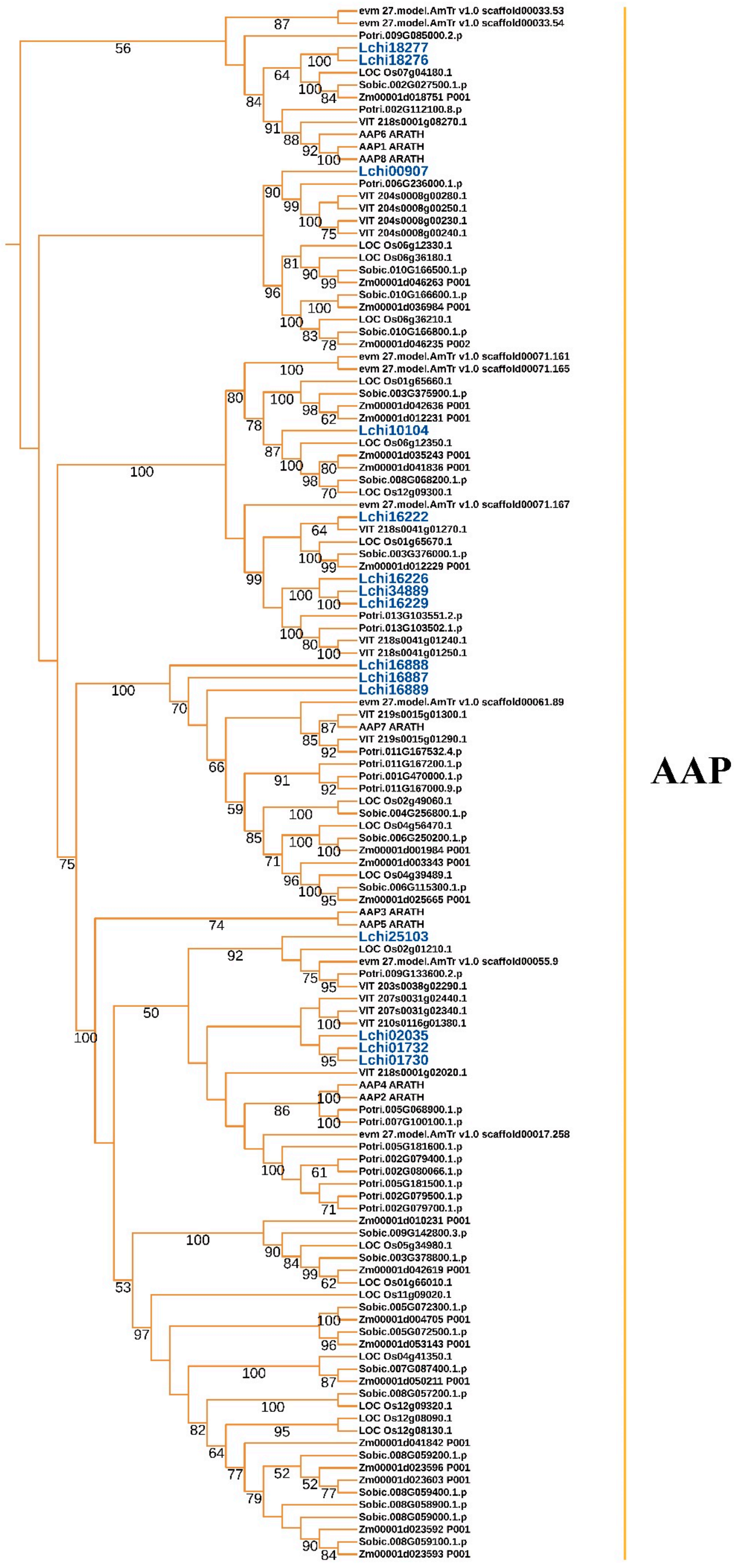
evm 27.model.AmTr v1.0 scaffold00033.53
evm 27.model.AmTr v1.0 scaffold00033.54
Potri.009G085000.2.p
Lchi18277
Lchi18276
LOC Os07g04180.1
Sobic.002G027500.1.p
Zm00001d018751 P001
Potri.002G112100.8.p
VIT 218s0001g08270.1
AAP6 ARATH
AAP1 ARATH
AAP8 ARATH
Lchi00907
Potri.006G236000.1.p
VIT 204s0008g00280.1
VIT 204s0008g00250.1
VIT 204s0008g00230.1
VIT 204s0008g00240.1
LOC Os06g12330.1
LOC Os06g36180.1
Sobic.010G166500.1.p
Zm00001d046263 P001
Sobic.010G166600.1.p
Zm00001d036984 P001
LOC Os06g36210.1
Sobic.010G166800.1.p
Zm00001d046235 P002
evm 27.model.AmTr v1.0 scaffold00071.161
evm 27.model.AmTr v1.0 scaffold00071.165
LOC Os01g65660.1
Sobic.003G375900.1.p
Zm00001d042636 P001
Zm00001d012231 P001
Lchi10104
LOC Os06g12350.1
Zm00001d035243 P001
Zm00001d041836 P001
Sobic.008G068200.1.p
LOC Os12g09300.1
evm 27.model.AmTr v1.0 scaffold00071.167
Lchi16222
VIT 218s0041g01270.1
LOC Os01g65670.1
Sobic.003G376000.1.p
Zm00001d012229 P001
Lchi16226
Lchi34889
Lchi16229
Potri.013G103551.2.p
Potri.013G103502.1.p
VIT 218s0041g01240.1
VIT 218s0041g01250.1
Lchi16888
Lchi16887
Lchi16889
evm 27.model.AmTr v1.0 scaffold00061.89
VIT 219s0015g01300.1
AAP7 ARATH
VIT 219s0015g01290.1
Potri.011G167532.4.p
Potri.011G167200.1.p
Potri.001G470000.1.p
Potri.011G167000.9.p
LOC Os02g49060.1
Sobic.004G256800.1.p
LOC Os04g56470.1
Sobic.006G250200.1.p
Zm00001d001984 P001
Zm00001d003343 P001
LOC Os04g39489.1
Sobic.006G115300.1.p
Zm00001d025665 P001
AAP3 ARATH
AAP5 ARATH
Lchi25103
LOC Os02g01210.1
evm 27.model.AmTr v1.0 scaffold00055.9
Potri.009G133600.2.p
VIT 203s0038g02290.1
VIT 207s0031g02440.1
VIT 207s0031g02340.1
VIT 210s0116g01380.1
Lchi02035
Lchi01732
Lchi01730
VIT 218s0001g02020.1
AAP4 ARATH
AAP2 ARATH
Potri.005G068900.1.p
Potri.007G100100.1.p
evm 27.model.AmTr v1.0 scaffold00017.258
Potri.005G181600.1.p
Potri.002G079400.1.p
Potri.002G080066.1.p
Potri.005G181500.1.p
Potri.002G079500.1.p
Potri.002G079700.1.p
Zm00001d010231 P001
Sobic.009G142800.3.p
LOC Os05g34980.1
Sobic.003G378800.1.p
Zm00001d042619 P001
LOC Os01g66010.1
LOC Os11g09020.1
Sobic.005G072300.1.p
Zm00001d004705 P001
Sobic.005G072500.1.p
Zm00001d053143 P001
LOC Os04g41350.1
Sobic.007G087400.1.p
Zm00001d050211 P001
Sobic.008G057200.1.p
LOC Os12g09320.1
LOC Os12g08090.1
LOC Os12g08130.1
Zm00001d041842 P001
Sobic.008G059200.1.p
Zm00001d023596 P001
Zm00001d023603 P001
Sobic.008G059400.1.p
Sobic.008G058900.1.p
Sobic.008G059000.1.p
Zm00001d023592 P001
Sobic.008G059100.1.p
Zm00001d023593 P001
AAP

c

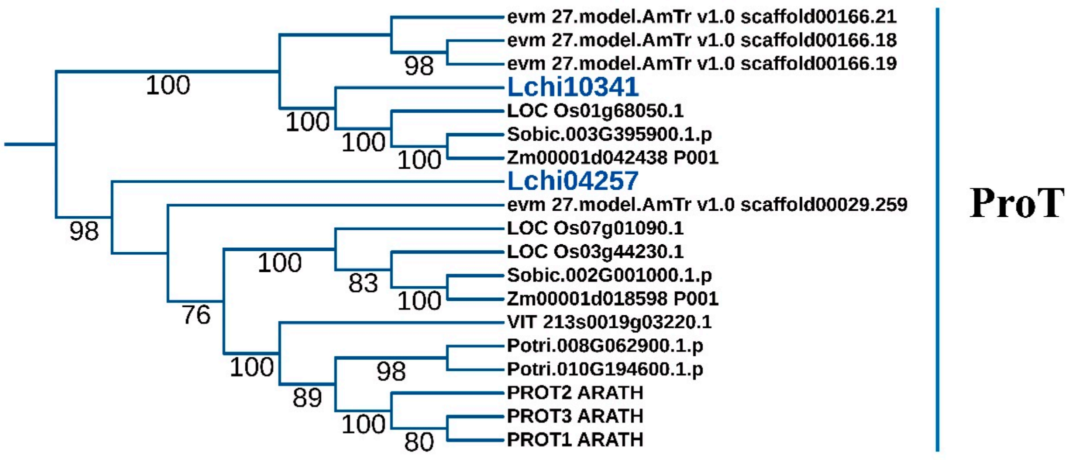
evm 27.model.AmTr v1.0 scaffold00166.21
evm 27.model.AmTr v1.0 scaffold00166.18
evm 27.model.AmTr v1.0 scaffold00166.19
Lchi10341
LOC Os01g68050.1
Sobic.003G395900.1.p
Zm00001d042438 P001
Lchi04257
evm 27.model.AmTr v1.0 scaffold00029.259
LOC Os07g01090.1
LOC Os03g44230.1
Sobic.002G001000.1.p
Zm00001d018598 P001
VIT 213s0019g03220.1
Potri.008G062900.1.p
Potri.010G194600.1.p
PROT2 ARATH
PROT3 ARATH
PROT1 ARATH
ProT

d

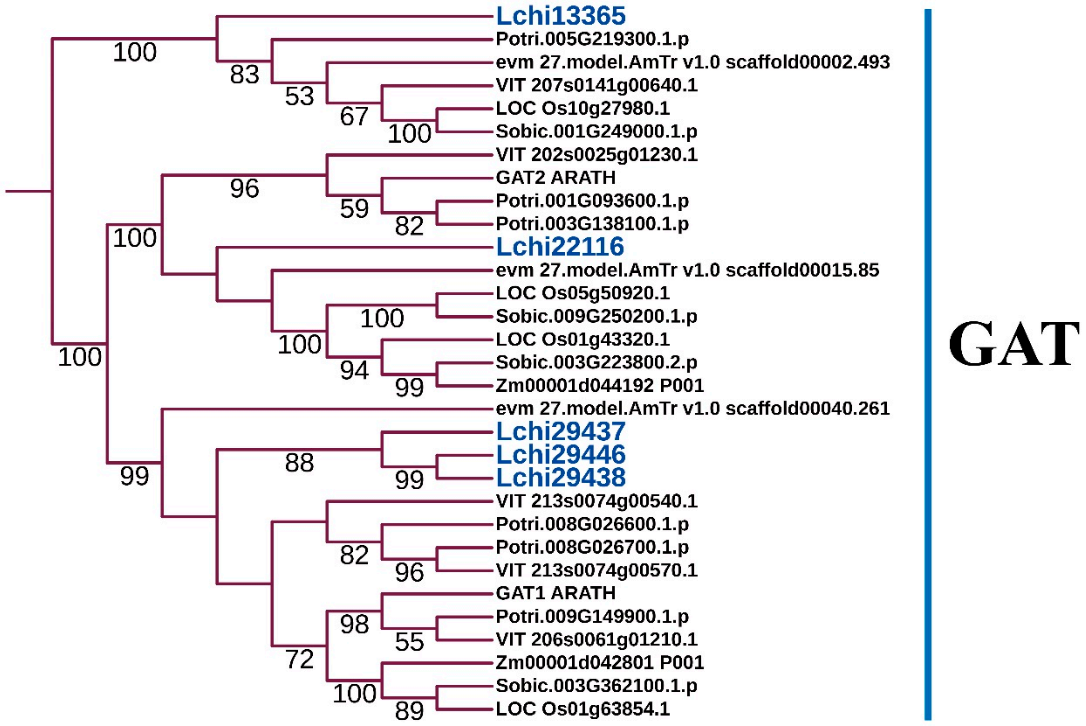
Lchi13365
Potri.005G219300.1.p
evm 27.model.AmTr v1.0 scaffold00002.493
VIT 207s0141g00640.1
LOC Os10g27980.1
Sobic.001G249000.1.p
VIT 202s0025g01230.1
GAT2 ARATH
Potri.001G093600.1.p
Potri.003G138100.1.p
Lchi22116
evm 27.model.AmTr v1.0 scaffold00015.85
LOC Os05g50920.1
Sobic.009G250200.1.p
LOC Os01g43320.1
Sobic.003G223800.2.p
Zm00001d044192 P001
evm 27.model.AmTr v1.0 scaffold00040.261
Lchi29437
Lchi29446
Lchi29438
VIT 213s0074g00540.1
Potri.008G026600.1.p
Potri.008G026700.1.p
VIT 213s0074g00570.1
GAT1 ARATH
Potri.009G149900.1.p
VIT 206s0061g01210.1
Zm00001d042801 P001
Sobic.003G362100.1.p
LOC Os01g63854.1
GAT

e

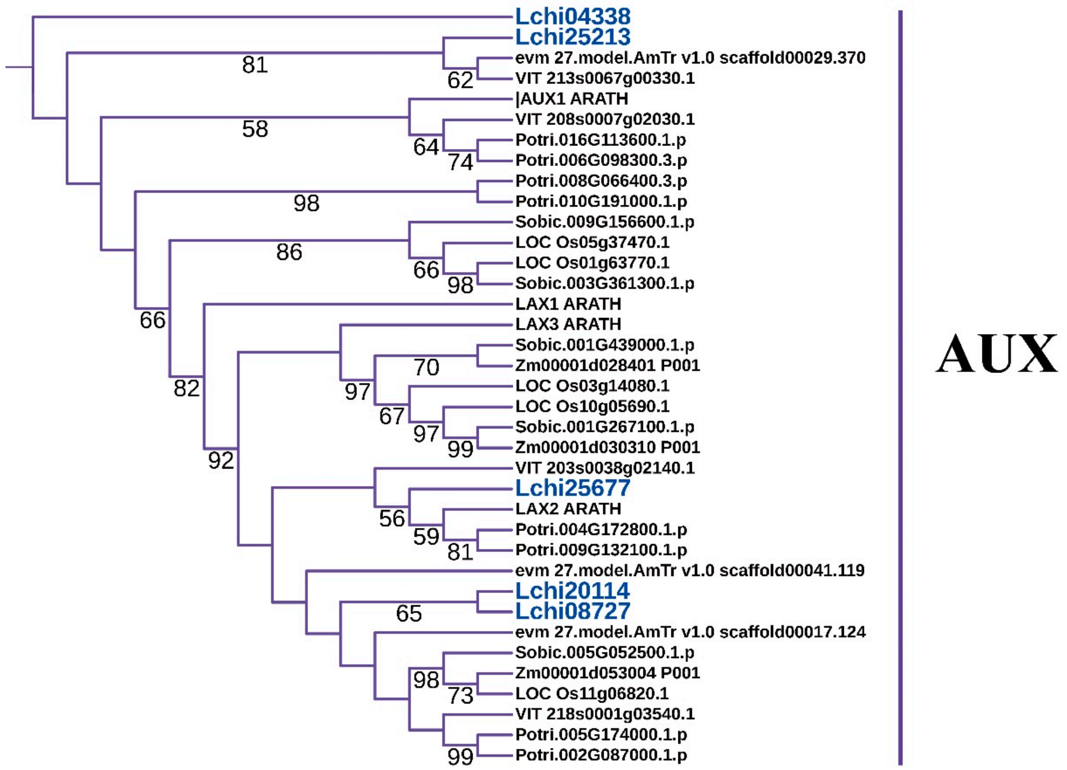
Lchi04338
Lchi25213
evm 27.model.AmTr v1.0 scaffold00029.370
VIT 213s0067g00330.1
|AUX1 ARATH
VIT 208s0007g02030.1
Potri.016G113600.1.p
Potri.006G098300.3.p
Potri.008G066400.3.p
Potri.010G191000.1.p
Sobic.009G156600.1.p
LOC Os05g37470.1
LOC Os01g63770.1
Sobic.003G361300.1.p
LAX1 ARATH
LAX3 ARATH
Sobic.001G439000.1.p
Zm00001d028401 P001
LOC Os03g14080.1
LOC Os10g05690.1
Sobic.001G267100.1.p
Zm00001d030310 P001
VIT 203s0038g02140.1
Lchi25677
LAX2 ARATH
Potri.004G172800.1.p
Potri.009G132100.1.p
evm 27.model.AmTr v1.0 scaffold00041.119
Lchi20114
Lchi08727
evm 27.model.AmTr v1.0 scaffold00017.124
Sobic.005G052500.1.p
Zm00001d053004 P001
LOC Os11g06820.1
VIT 218s0001g03540.1
Potri.005G174000.1.p
Potri.002G087000.1.p
81
62
58
64
74
98
86
66
98
66
70
82
97
67
97
99
92
56
59
81
65
98
73
99
AUX

f

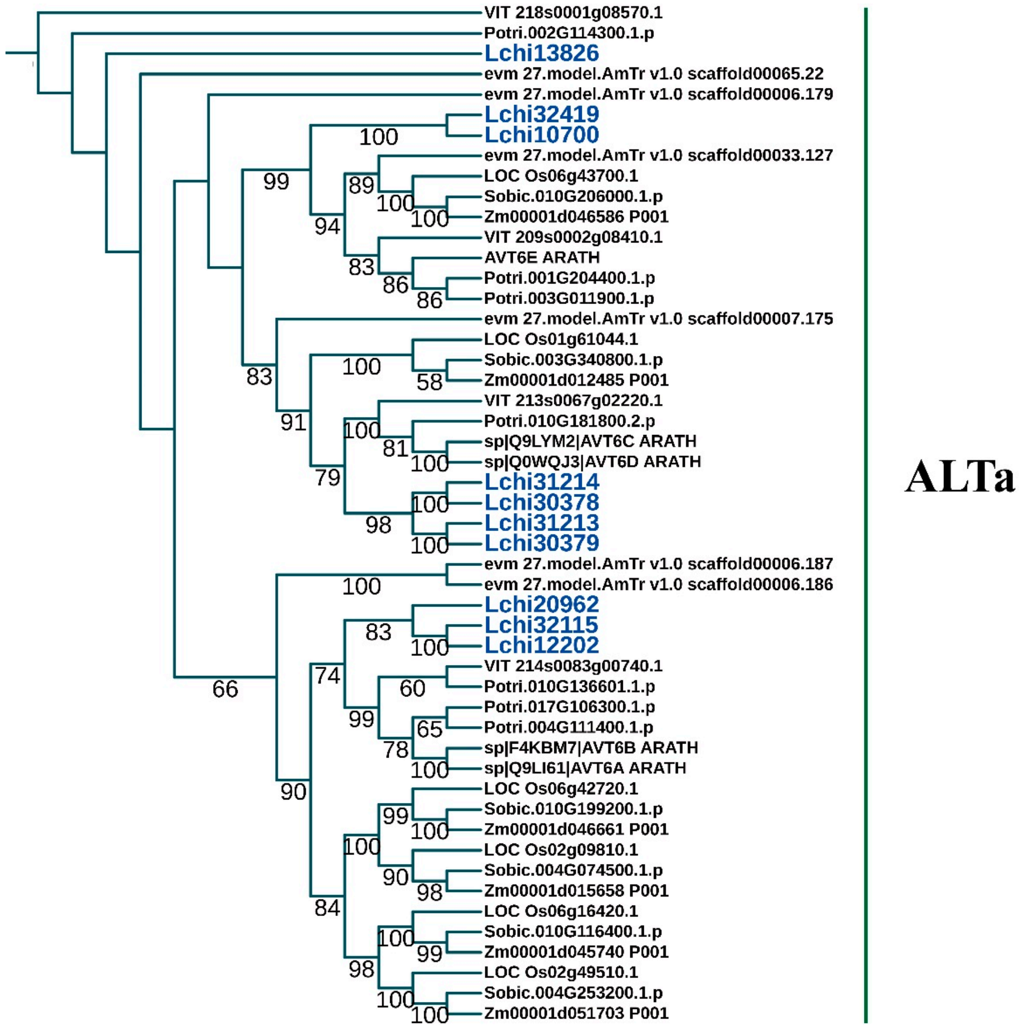
VIT 218s0001g08570.1
Potri.002G114300.1.p
Lchi13826
evm 27.model.AmTr v1.0 scaffold00065.22
evm 27.model.AmTr v1.0 scaffold00006.179
Lchi32419
Lchi10700
evm 27.model.AmTr v1.0 scaffold00033.127
LOC Os06g43700.1
Sobic.010G206000.1.p
Zm00001d046586 P001
VIT 209s0002g08410.1
AVT6E ARATH
Potri.001G204400.1.p
Potri.003G011900.1.p
evm 27.model.AmTr v1.0 scaffold00007.175
LOC Os01g61044.1
Sobic.003G340800.1.p
Zm00001d012485 P001
VIT 213s0067g02220.1
Potri.010G181800.2.p
sp|Q9LYM2|AVT6C ARATH
sp|Q0WQJ3|AVT6D ARATH
Lchi31214
Lchi30378
Lchi31213
Lchi30379
evm 27.model.AmTr v1.0 scaffold00006.187
evm 27.model.AmTr v1.0 scaffold00006.186
Lchi20962
Lchi32115
Lchi12202
VIT 214s0083g00740.1
Potri.010G136601.1.p
Potri.017G106300.1.p
Potri.004G111400.1.p
sp|F4KBM7|AVT6B ARATH
sp|Q9LI61|AVT6A ARATH
LOC Os06g42720.1
Sobic.010G199200.1.p
Zm00001d046661 P001
LOC Os02g09810.1
Sobic.004G074500.1.p
Zm00001d015658 P001
LOC Os06g16420.1
Sobic.010G116400.1.p
Zm00001d045740 P001
LOC Os02g49510.1
Sobic.004G253200.1.p
Zm00001d051703 P001
ALTa

g

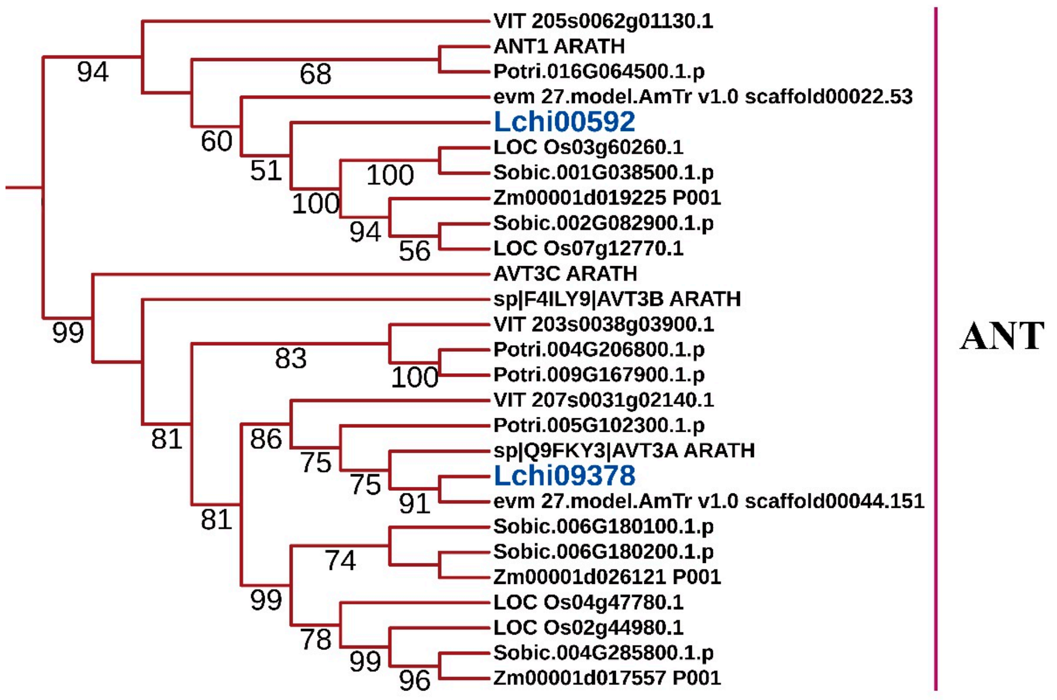
VIT 205s0062g01130.1
ANT1 ARATH
Potri.016G064500.1.p
evm 27.model.AmTr v1.0 scaffold00022.53
Lchi00592
LOC Os03g60260.1
Sobic.001G038500.1.p
Zm00001d019225 P001
Sobic.002G082900.1.p
LOC Os07g12770.1
AVT3C ARATH
sp|F4ILY9|AVT3B ARATH
VIT 203s0038g03900.1
Potri.004G206800.1.p
Potri.009G167900.1.p
VIT 207s0031g02140.1
Potri.005G102300.1.p
sp|Q9FKY3|AVT3A ARATH
Lchi09378
evm 27.model.AmTr v1.0 scaffold00044.151
Sobic.006G180100.1.p
Sobic.006G180200.1.p
Zm00001d026121 P001
LOC Os04g47780.1
LOC Os02g44980.1
Sobic.004G285800.1.p
Zm00001d017557 P001
ANT

h

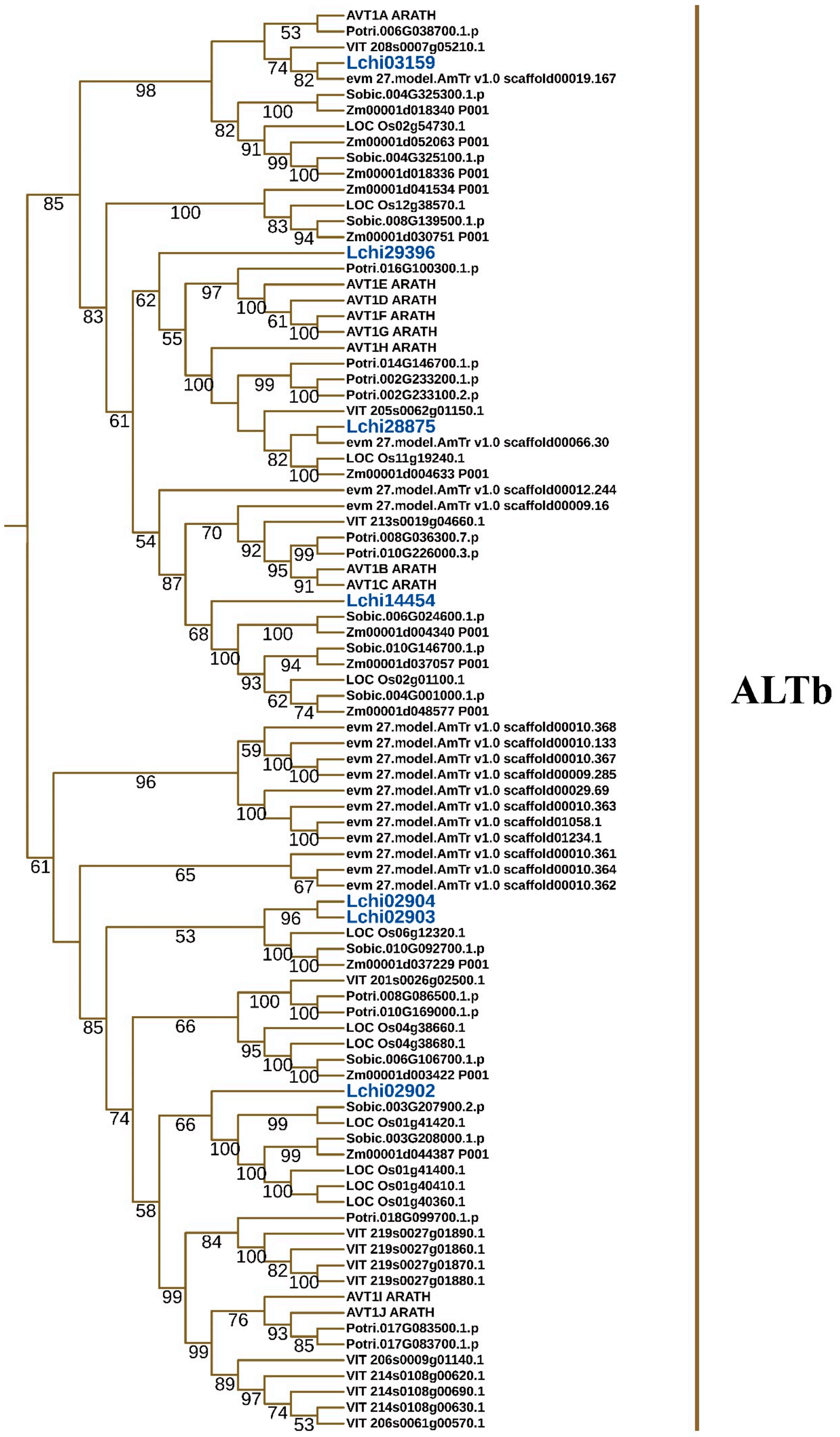
AVT1A ARATH
Potri.006G038700.1.p
VIT 208s0007g05210.1
Lchi03159
evm 27.model.AmTr v1.0 scaffold00019.167
Sobic.004G325300.1.p
Zm00001d018340 P001
LOC Os02g54730.1
Zm00001d052063 P001
Sobic.004G325100.1.p
Zm00001d018336 P001
Zm00001d041534 P001
LOC Os12g38570.1
Sobic.008G139500.1.p
Zm00001d030751 P001
Lchi29396
Potri.016G100300.1.p
AVT1E ARATH
AVT1D ARATH
AVT1F ARATH
AVT1G ARATH
AVT1H ARATH
Potri.014G146700.1.p
Potri.002G233200.1.p
Potri.002G233100.2.p
VIT 205s0062g01150.1
Lchi28875
evm 27.model.AmTr v1.0 scaffold00066.30
LOC Os11g19240.1
Zm00001d004633 P001
evm 27.model.AmTr v1.0 scaffold00012.244
evm 27.model.AmTr v1.0 scaffold00009.16
VIT 213s0019g04660.1
Potri.008G036300.7.p
Potri.010G226000.3.p
AVT1B ARATH
AVT1C ARATH
Lchi14454
Sobic.006G024600.1.p
Zm00001d004340 P001
Sobic.010G146700.1.p
Zm00001d037057 P001
LOC Os02g01100.1
Sobic.004G001000.1.p
Zm00001d048577 P001
evm 27.model.AmTr v1.0 scaffold00010.368
evm 27.model.AmTr v1.0 scaffold00010.133
evm 27.model.AmTr v1.0 scaffold00010.367
evm 27.model.AmTr v1.0 scaffold00009.285
evm 27.model.AmTr v1.0 scaffold00029.69
evm 27.model.AmTr v1.0 scaffold00010.363
evm 27.model.AmTr v1.0 scaffold01058.1
evm 27.model.AmTr v1.0 scaffold01234.1
evm 27.model.AmTr v1.0 scaffold00010.361
evm 27.model.AmTr v1.0 scaffold00010.364
evm 27.model.AmTr v1.0 scaffold00010.362
Lchi02904
Lchi02903
LOC Os06g12320.1
Sobic.010G092700.1.p
Zm00001d037229 P001
VIT 201s0026g02500.1
Potri.008G086500.1.p
Potri.010G169000.1.p
LOC Os04g38660.1
LOC Os04g38680.1
Sobic.006G106700.1.p
Zm00001d003422 P001
Lchi02902
Sobic.003G207900.2.p
LOC Os01g41420.1
Sobic.003G208000.1.p
Zm00001d044387 P001
LOC Os01g41400.1
LOC Os01g40410.1
LOC Os01g40360.1
Potri.018G099700.1.p
VIT 219s0027g01890.1
VIT 219s0027g01860.1
VIT 219s0027g01870.1
VIT 219s0027g01880.1
AVT1I ARATH
AVT1J ARATH
Potri.017G083500.1.p
Potri.017G083700.1.p
VIT 206s0009g01140.1
VIT 214s0108g00620.1
VIT 214s0108g00690.1
VIT 214s0108g00630.1
VIT 206s0061g00570.1
ALTb

Supplementary Figure S1. Phylogenetic relationships of *L. chinense* (Lc), *O. sativa* (Os), *A. thaliana* (At), *A. trichopoda* (Atr), *S. bicolor* (Sb), *P. trichocarpa* (Pt), *Z. mays* (Zm), and *V. vinifera* (Vv) AAAP proteins. Multiple sequence alignment of full-length proteins was performed by muscle and the phylogenetic tree using the maximum-likelihood estimation with IQtree2.13 and 1000 bootstrap replications: the optimal model was JTT+F+R7. The tree was divided into 8 subgroups (a-h), marked by different-colored backgrounds.
