# Supplementary material for: Identification, Phylogenetic and Expression Analyses of the AAAP Gene Family in Liriodendron chinense Reveal Their Putative Functions in Response to Organ and Multiple Abiotic Stresses
Source: Int J Mol Sci. 2022 Apr 26;23(9):4765. doi: 10.3390/ijms23094765 (PMC9100865; doi:10.3390/ijms23094765)
Supplement: Supplementary file 1 [file ijms-23-04765-s001.zip › Table S1.pdf]

Supplementary Table S1 The information of the all-protein sequences

Species	Gene ID	Gene Subgrops	Gene Name	Data Sources
Amborella trichopoda	evm_27.model.AmTr_v1.0_scaffold00001.289	LHT		Phytozome 13
	evm_27.model.AmTr_v1.0_scaffold00002.493	GAT		Phytozome 13
	evm_27.model.AmTr_v1.0_scaffold00005.76	LHT		Phytozome 13
	evm_27.model.AmTr_v1.0_scaffold00006.179	ATLa		Phytozome 13
	evm_27.model.AmTr_v1.0_scaffold00006.186	ATLa		Phytozome 13
	evm_27.model.AmTr_v1.0_scaffold00006.187	ATLa		Phytozome 13
	evm_27.model.AmTr_v1.0_scaffold00007.175	ATLa		Phytozome 13
	evm_27.model.AmTr_v1.0_scaffold00009.16	ATLb		Phytozome 13
	evm_27.model.AmTr_v1.0_scaffold00009.285	ATLb		Phytozome 13
	evm_27.model.AmTr_v1.0_scaffold00010.133	ATLb		Phytozome 13
	evm_27.model.AmTr_v1.0_scaffold00010.361	ATLb		Phytozome 13
	evm_27.model.AmTr_v1.0_scaffold00010.362	ATLb		Phytozome 13
	evm_27.model.AmTr_v1.0_scaffold00010.363	ATLb		Phytozome 13
	evm_27.model.AmTr_v1.0_scaffold00010.364	ATLb		Phytozome 13
	evm_27.model.AmTr_v1.0_scaffold00010.367	ATLb		Phytozome 13
	evm_27.model.AmTr_v1.0_scaffold00010.368	ATLb		Phytozome 13
	evm_27.model.AmTr_v1.0_scaffold00012.244	ATLb		Phytozome 13
	evm_27.model.AmTr_v1.0_scaffold00015.85	GAT		Phytozome 13
	evm_27.model.AmTr_v1.0_scaffold00017.124	AUX		Phytozome 13
	evm_27.model.AmTr_v1.0_scaffold00017.258	APP		Phytozome 13
	evm_27.model.AmTr_v1.0_scaffo	ATLb		Phytozome 13

ld00019.167		
evm_27.model.AmTr_v1.0_scaffold00022.53	ANT	Phytozome 13
evm_27.model.AmTr_v1.0_scaffold00029.259	PorT	Phytozome 13
evm_27.model.AmTr_v1.0_scaffold00029.370	AUX	Phytozome 13
evm_27.model.AmTr_v1.0_scaffold00029.69	ATLb	Phytozome 13
evm_27.model.AmTr_v1.0_scaffold00033.127	ATLa	Phytozome 13
evm_27.model.AmTr_v1.0_scaffold00033.53	APP	Phytozome 13
evm_27.model.AmTr_v1.0_scaffold00033.54	APP	Phytozome 13
evm_27.model.AmTr_v1.0_scaffold00040.261	GAT	Phytozome 13
evm_27.model.AmTr_v1.0_scaffold00041.119	AUX	Phytozome 13
evm_27.model.AmTr_v1.0_scaffold00044.151	ANT	Phytozome 13
evm_27.model.AmTr_v1.0_scaffold00055.9	APP	Phytozome 13
evm_27.model.AmTr_v1.0_scaffold00059.251	LHT	Phytozome 13
evm_27.model.AmTr_v1.0_scaffold00061.89	APP	Phytozome 13
evm_27.model.AmTr_v1.0_scaffold00065.22	ATLa	Phytozome 13
evm_27.model.AmTr_v1.0_scaffold00066.30	ATLb	Phytozome 13
evm_27.model.AmTr_v1.0_scaffold00071.161	APP	Phytozome 13
evm_27.model.AmTr_v1.0_scaffold00071.165	APP	Phytozome 13
evm_27.model.AmTr_v1.0_scaffold00071.167	APP	Phytozome 13
evm_27.model.AmTr_v1.0_scaffold00166.18	PorT	Phytozome 13
evm_27.model.AmTr_v1.0_scaffold00166.19	PorT	Phytozome 13
evm_27.model.AmTr_v1.0_scaffold00166.21	PorT	Phytozome 13
evm_27.model.AmTr_v1.0_scaffo	ATLb	Phytozome 13

	ld01058.1			
	evm_27.model.AmTr_v1.0_scaffold01234.1	ATLb		Phytozome 13
Liriodendron chinense	Lchi00592	ANT	*LcANT1*	L. chinense genome
	Lchi00907	APP	*LcAPP2*	L. chinense genome
	Lchi01730	APP	*LcAPP4b*	L. chinense genome
	Lchi01732	APP	*LcAPP9*	L. chinense genome
	Lchi02035	APP	*LcAPP4a*	L. chinense genome
	Lchi02902	ATLb	*LcATL15*	L. chinense genome
	Lchi02903	ATLb	*LcATL12a*	L. chinense genome
	Lchi02904	ATLb	*LcATL12b*	L. chinense genome
	Lchi03159	ATLb	*LcATL9*	L. chinense genome
	Lchi04257	PorT	*LcPorT2*	L. chinense genome
	Lchi04338	AUX	*LcAUX1a*	L. chinense genome
	Lchi08727	AUX	*LcLHT4*	L. chinense genome
	Lchi09378	ANT	*LcLAX5a*	L. chinense genome
	Lchi09528	LHT	*LcANT2*	L. chinense genome
	Lchi10104	APP	*LcAPP17*	L. chinense genome
	Lchi10219	LHT	*LcLHT1*	L. chinense genome
	Lchi10341	PorT	*LcPorT1*	L. chinense genome
	Lchi10700	ATLa	*LcATL1a*	L. chinense genome
	Lchi12202	ATLa	*LcATL5c*	L. chinense genome
	Lchi13365	GAT	*LcGAT4*	L. chinense genome
	Lchi13826	ATLa	*LcAVT6*	L. chinense

			genome
Lchi13928	LHT	*LcLHT8*	L. chinense genome
Lchi14454	ATLb	*LcATL11*	L. chinense genome
Lchi16175	LHT	*LcLHT3*	L. chinense genome
Lchi16222	APP	*LcAPP6a*	L. chinense genome
Lchi16226	APP	*LcAPP6b*	L. chinense genome
Lchi16229	APP	*LcAPP6d*	L. chinense genome
Lchi16887	APP	*LcAPP7c*	L. chinense genome
Lchi16888	APP	*LcAPP7a*	L. chinense genome
Lchi16889	APP	*LcAPP7b*	L. chinense genome
Lchi18276	APP	*LcAPP1a*	L. chinense genome
Lchi18277	APP	*LcAPP1b*	L. chinense genome
Lchi19559	LHT	*LcLHT2*	L. chinense genome
Lchi20114	AUX	*LcLAX5b*	L. chinense genome
Lchi20962	ATLa	*LcATL5a*	L. chinense genome
Lchi22116	GAT	*LcGAT2*	L. chinense genome
Lchi25103	APP	*LcAPP4c*	L. chinense genome
Lchi25213	AUX	*LcAUX1b*	L. chinense genome
Lchi25677	AUX	*LcLAX2*	L. chinense genome
Lchi28875	ATLb	*LcATL8a*	L. chinense genome
Lchi29396	ATLb	*LcATL8b*	L. chinense genome
Lchi29437	GAT	*LcGAT1a*	L. chinense genome
Lchi29438	GAT	*LcGAT1*	L. chinense

			c	genome
	Lchi29446	GAT	*LcGAT1b*	L. chinense genome
	Lchi30378	ATLa	*LcATL7c*	L. chinense genome
	Lchi30379	ATLa	*LcATL7a*	L. chinense genome
	Lchi31213	ATLa	*LcATL7b*	L. chinense genome
	Lchi31214	ATLa	*LcATL7d*	L. chinense genome
	Lchi32115	ATLa	*LcATL5b*	L. chinense genome
	Lchi32419	ATLa	*LcATL1b*	L. chinense genome
	Lchi33844	LHT	*LcLHT6*	L. chinense genome
	Lchi34889	APP	*LcAPP6c*	L. chinense genome
Oryza sativa	LOC_Os01g40360.1	ATLb		Phytozome 13
	LOC_Os01g40410.1	ATLb		Phytozome 13
	LOC_Os01g41400.1	ATLb		Phytozome 13
	LOC_Os01g41420.1	ATLb		Phytozome 13
	LOC_Os01g43320.1	GAT		Phytozome 13
	LOC_Os01g61044.1	ATLa		Phytozome 13
	LOC_Os01g63770.1	AUX		Phytozome 13
	LOC_Os01g63854.1	GAT		Phytozome 13
	LOC_Os01g65660.1	APP		Phytozome 13
	LOC_Os01g65670.1	APP		Phytozome 13
	LOC_Os01g66010.1	APP		Phytozome 13
	LOC_Os01g68050.1	PorT		Phytozome 13
	LOC_Os02g01100.1	ATLb		Phytozome 13
	LOC_Os02g01210.1	APP		Phytozome 13
	LOC_Os02g09810.1	ATLa		Phytozome 13
	LOC_Os02g44980.1	ANT		Phytozome 13
	LOC_Os02g49060.1	APP		Phytozome 13
	LOC_Os02g49510.1	ATLa		Phytozome 13
	LOC_Os02g54730.1	ATLb		Phytozome 13
	LOC_Os03g14080.1	AUX		Phytozome 13
	LOC_Os03g44230.1	PorT		Phytozome 13
	LOC_Os03g60260.1	ANT		Phytozome 13
	LOC_Os04g38660.1	ATLb		Phytozome 13
	LOC_Os04g38680.1	ATLb		Phytozome 13
	LOC_Os04g38860.1	LHT		Phytozome 13

	LOC_Os04g39489.1	APP	Phytozome 13
	LOC_Os04g41350.1	APP	Phytozome 13
	LOC_Os04g47420.1	LHT	Phytozome 13
	LOC_Os04g47780.1	ANT	Phytozome 13
	LOC_Os04g56470.1	APP	Phytozome 13
	LOC_Os05g14820.1	LHT	Phytozome 13
	LOC_Os05g34980.1	APP	Phytozome 13
	LOC_Os05g37470.1	AUX	Phytozome 13
	LOC_Os05g50920.1	GAT	Phytozome 13
	LOC_Os06g12320.1	ATLb	Phytozome 13
	LOC_Os06g12330.1	APP	Phytozome 13
	LOC_Os06g12350.1	APP	Phytozome 13
	LOC_Os06g16420.1	ATLa	Phytozome 13
	LOC_Os06g36180.1	APP	Phytozome 13
	LOC_Os06g36210.1	APP	Phytozome 13
	LOC_Os06g42720.1	ATLa	Phytozome 13
	LOC_Os06g43700.1	ATLa	Phytozome 13
	LOC_Os07g01090.1	PorT	Phytozome 13
	LOC_Os07g04180.1	APP	Phytozome 13
	LOC_Os07g12770.1	ANT	Phytozome 13
	LOC_Os08g03350.1	LHT	Phytozome 13
	LOC_Os10g05690.1	AUX	Phytozome 13
	LOC_Os10g27980.1	GAT	Phytozome 13
	LOC_Os11g06820.1	AUX	Phytozome 13
	LOC_Os11g09020.1	APP	Phytozome 13
	LOC_Os11g19240.1	ATLb	Phytozome 13
	LOC_Os12g08090.1	APP	Phytozome 13
	LOC_Os12g08130.1	APP	Phytozome 13
	LOC_Os12g09300.1	APP	Phytozome 13
	LOC_Os12g09320.1	APP	Phytozome 13
	LOC_Os12g14100.1	LHT	Phytozome 13
	LOC_Os12g30040.1	LHT	Phytozome 13
	LOC_Os12g38570.1	ATLb	Phytozome 13
Populus trichocarpa	Potri.001G093600.1.p	GAT	Phytozome 13
	Potri.001G204400.1.p	ATLa	Phytozome 13
	Potri.001G335200.1.p	LHT	Phytozome 13
	Potri.001G335300.1.p	LHT	Phytozome 13
	Potri.001G470000.1.p	APP	Phytozome 13
	Potri.002G012900.1.p	LHT	Phytozome 13
	Potri.002G079400.1.p	APP	Phytozome 13
	Potri.002G079500.1.p	APP	Phytozome 13
	Potri.002G079700.1.p	APP	Phytozome 13
	Potri.002G080066.1.p	APP	Phytozome 13
	Potri.002G087000.1.p	AUX	Phytozome 13

Potri.002G112100.8.p	APP	Phytozome 13
Potri.002G114300.1.p	ATLa	Phytozome 13
Potri.002G233100.2.p	ATLb	Phytozome 13
Potri.002G233200.1.p	ATLb	Phytozome 13
Potri.003G011900.1.p	ATLa	Phytozome 13
Potri.003G138100.1.p	GAT	Phytozome 13
Potri.004G111400.1.p	ATLa	Phytozome 13
Potri.004G172800.1.p	AUX	Phytozome 13
Potri.004G181000.1.p	LHT	Phytozome 13
Potri.004G181100.1.p	LHT	Phytozome 13
Potri.004G181200.1.p	LHT	Phytozome 13
Potri.004G206800.1.p	ANT	Phytozome 13
Potri.005G068900.1.p	APP	Phytozome 13
Potri.005G102300.1.p	ANT	Phytozome 13
Potri.005G174000.1.p	AUX	Phytozome 13
Potri.005G181500.1.p	APP	Phytozome 13
Potri.005G181600.1.p	APP	Phytozome 13
Potri.005G219300.1.p	GAT	Phytozome 13
Potri.006G038700.1.p	ATLb	Phytozome 13
Potri.006G098300.3.p	AUX	Phytozome 13
Potri.006G236000.1.p	APP	Phytozome 13
Potri.007G100100.1.p	APP	Phytozome 13
Potri.008G026600.1.p	GAT	Phytozome 13
Potri.008G026700.1.p	GAT	Phytozome 13
Potri.008G036300.7.p	ATLb	Phytozome 13
Potri.008G062900.1.p	PorT	Phytozome 13
Potri.008G066400.3.p	AUX	Phytozome 13
Potri.008G086500.1.p	ATLb	Phytozome 13
Potri.008G118000.1.p	LHT	Phytozome 13
Potri.008G179000.1.p	LHT	Phytozome 13
Potri.009G085000.2.p	APP	Phytozome 13
Potri.009G132100.1.p	AUX	Phytozome 13
Potri.009G133600.2.p	APP	Phytozome 13
Potri.009G140800.1.p	LHT	Phytozome 13
Potri.009G149900.1.p	GAT	Phytozome 13
Potri.009G167900.1.p	ANT	Phytozome 13
Potri.010G055800.1.p	LHT	Phytozome 13
Potri.010G128300.1.p	LHT	Phytozome 13
Potri.010G136601.1.p	ATLa	Phytozome 13
Potri.010G169000.1.p	ATLb	Phytozome 13
Potri.010G181800.2.p	ATLa	Phytozome 13
Potri.010G191000.1.p	AUX	Phytozome 13
Potri.010G194600.1.p	PorT	Phytozome 13
Potri.010G226000.3.p	ATLb	Phytozome 13

	Potri.011G167000.9.p	APP	Phytozome 13
	Potri.011G167200.1.p	APP	Phytozome 13
	Potri.011G167532.4.p	APP	Phytozome 13
	Potri.013G103502.1.p	APP	Phytozome 13
	Potri.013G103551.2.p	APP	Phytozome 13
	Potri.014G036500.1.p	LHT	Phytozome 13
	Potri.014G146700.1.p	ATLb	Phytozome 13
	Potri.015G091600.1.p	LHT	Phytozome 13
	Potri.016G064500.1.p	ANT	Phytozome 13
	Potri.016G100300.1.p	ATLb	Phytozome 13
	Potri.016G113600.1.p	AUX	Phytozome 13
	Potri.017G083500.1.p	ATLb	Phytozome 13
	Potri.017G083700.1.p	ATLb	Phytozome 13
	Potri.017G106300.1.p	ATLa	Phytozome 13
	Potri.018G099700.1.p	ATLb	Phytozome 13
	Sobic.001G038500.1.p	ANT	Phytozome 13
	Sobic.001G249000.1.p	GAT	Phytozome 13
	Sobic.001G267100.1.p	AUX	Phytozome 13
	Sobic.001G439000.1.p	AUX	Phytozome 13
	Sobic.002G001000.1.p	PorT	Phytozome 13
	Sobic.002G027500.1.p	APP	Phytozome 13
	Sobic.002G082900.1.p	ANT	Phytozome 13
	Sobic.003G207900.2.p	ATLb	Phytozome 13
	Sobic.003G208000.1.p	ATLb	Phytozome 13
	Sobic.003G223800.2.p	GAT	Phytozome 13
	Sobic.003G340800.1.p	ATLa	Phytozome 13
	Sobic.003G361300.1.p	AUX	Phytozome 13
	Sobic.003G362100.1.p	GAT	Phytozome 13
	Sobic.003G375900.1.p	APP	Phytozome 13
Sorghum bicolor	Sobic.003G376000.1.p	APP	Phytozome 13
	Sobic.003G378800.1.p	APP	Phytozome 13
	Sobic.003G395900.1.p	PorT	Phytozome 13
	Sobic.004G001000.1.p	ATLb	Phytozome 13
	Sobic.004G074500.1.p	ATLa	Phytozome 13
	Sobic.004G253200.1.p	ATLa	Phytozome 13
	Sobic.004G256800.1.p	APP	Phytozome 13
	Sobic.004G285800.1.p	ANT	Phytozome 13
	Sobic.004G306900.1.p	LHT	Phytozome 13
	Sobic.004G325100.1.p	ATLb	Phytozome 13
	Sobic.004G325300.1.p	ATLb	Phytozome 13
	Sobic.005G052500.1.p	AUX	Phytozome 13
	Sobic.005G072300.1.p	APP	Phytozome 13
	Sobic.005G072500.1.p	APP	Phytozome 13
	Sobic.006G024600.1.p	ATLb	Phytozome 13

	Sobic.006G106700.1.p	ATLb	Phytozome 13
	Sobic.006G108300.1.p	LHT	Phytozome 13
	Sobic.006G115300.1.p	APP	Phytozome 13
	Sobic.006G178500.1.p	LHT	Phytozome 13
	Sobic.006G180100.1.p	ANT	Phytozome 13
	Sobic.006G180200.1.p	ANT	Phytozome 13
	Sobic.006G250200.1.p	APP	Phytozome 13
	Sobic.007G025500.1.p	LHT	Phytozome 13
	Sobic.007G025600.2.p	LHT	Phytozome 13
	Sobic.007G025700.1.p	LHT	Phytozome 13
	Sobic.007G025800.1.p	LHT	Phytozome 13
	Sobic.007G025900.1.p	LHT	Phytozome 13
	Sobic.007G087400.1.p	APP	Phytozome 13
	Sobic.007G092400.1.p	LHT	Phytozome 13
	Sobic.008G057200.1.p	APP	Phytozome 13
	Sobic.008G058900.1.p	APP	Phytozome 13
	Sobic.008G059000.1.p	APP	Phytozome 13
	Sobic.008G059100.1.p	APP	Phytozome 13
	Sobic.008G059200.1.p	APP	Phytozome 13
	Sobic.008G059400.1.p	APP	Phytozome 13
	Sobic.008G068200.1.p	APP	Phytozome 13
	Sobic.008G088500.1.p	LHT	Phytozome 13
	Sobic.008G097700.1.p	LHT	Phytozome 13
	Sobic.008G139500.1.p	ATLb	Phytozome 13
	Sobic.009G087900.1.p	LHT	Phytozome 13
	Sobic.009G095640.1.p	LHT	Phytozome 13
	Sobic.009G142800.3.p	APP	Phytozome 13
	Sobic.009G156600.1.p	AUX	Phytozome 13
	Sobic.009G250200.1.p	GAT	Phytozome 13
	Sobic.010G092700.1.p	ATLb	Phytozome 13
	Sobic.010G116400.1.p	ATLa	Phytozome 13
	Sobic.010G146700.1.p	ATLb	Phytozome 13
	Sobic.010G166500.1.p	APP	Phytozome 13
	Sobic.010G166600.1.p	APP	Phytozome 13
	Sobic.010G166800.1.p	APP	Phytozome 13
	Sobic.010G199200.1.p	ATLa	Phytozome 13
	Sobic.010G206000.1.p	ATLa	Phytozome 13
Arabidopsis thaliana	sp|F4HW02|GAT1_ARATH	GAT	uniport
	sp|F4ILY9|AVT3B_ARATH	ANT	uniport
	sp|F4IUW3|AVT1C_ARATH	ATLb	uniport
	sp|F4IZW8|AVT1F_ARATH	ATLb	uniport
	sp|F4J1Q9|AVT1I_ARATH	ATLb	uniport
	sp|F4JE35|AVT1B_ARATH	ATLb	uniport
	sp|F4KBM7|AVT6B_ARATH	ATLa	uniport

	sp|O22719|LHTL3_ARATH	LHT	uniport
	sp|O80592|AAP8_ARATH	APP	uniport
	sp|O80668|AVT1A_ARATH	ATLb	uniport
	sp|P92934|AAP6_ARATH	APP	uniport
	sp|P92961|PROT1_ARATH	PorT	uniport
	sp|P92962|PROT2_ARATH	PorT	uniport
	sp|Q0WQJ3|AVT6D_ARATH	ATLa	uniport
	sp|Q1PER9|AVT1G_ARATH	ATLb	uniport
	sp|Q38967|AAP2_ARATH	APP	uniport
	sp|Q39134|AAP3_ARATH	APP	uniport
	sp|Q42400|AAP1_ARATH	APP	uniport
	sp|Q84WE9|LHTL7_ARATH	LHT	uniport
	sp|Q8GUM3|AAP5_ARATH	APP	uniport
	sp|Q8GYS4|AVT1D_ARATH	ATLb	uniport
	sp|Q8L4X4|GAT2_ARATH	GAT	uniport
	sp|Q8LPF4|AVT1E_ARATH	ATLb	uniport
	sp|Q96247|AUX1_ARATH	AUX	uniport
	sp|Q9C6M2|LHTL6_ARATH	LHT	uniport
	sp|Q9C733|LHTL1_ARATH	LHT	uniport
	sp|Q9C9J0|LHTL5_ARATH	LHT	uniport
	sp|Q9CA25|LAX3_ARATH	AUX	uniport
	sp|Q9FF99|AAP7_ARATH	APP	uniport
	sp|Q9FKS8|LHT1_ARATH	LHT	uniport
	sp|Q9FKY3|AVT3A_ARATH	ANT	uniport
	sp|Q9FN04|AAP4_ARATH	APP	uniport
	sp|Q9LFB2|LAX1_ARATH	AUX	uniport
	sp|Q9LFE3|AVT1H_ARATH	ATLb	uniport
	sp|Q9LI61|AVT6A_ARATH	ATLa	uniport
	sp|Q9LRB5|LHT2_ARATH	LHT	uniport
	sp|Q9LXF8|AVT1J_ARATH	ATLb	uniport
	sp|Q9LYM2|AVT6C_ARATH	ATLa	uniport
	sp|Q9M8L9|AVT6E_ARATH	ATLa	uniport
	sp|Q9S836|LAX2_ARATH	AUX	uniport
	sp|Q9SF09|ANT1_ARATH	ANT	uniport
	sp|Q9SJP9|PROT3_ARATH	PorT	uniport
	sp|Q9SR44|LHTL2_ARATH	LHT	uniport
	sp|Q9SS86|LHTL4_ARATH	LHT	uniport
	sp|Q9SVG0|AVT3C_ARATH	ANT	uniport
	sp|Q9SX98|LHTL8_ARATH	LHT	uniport
	VIT_200s0302g00020.1	LHT	Phytozome 13
	VIT_201s0010g01490.1	LHT	Phytozome 13
Vitis vinifera	VIT_201s0010g02490.1	LHT	Phytozome 13
	VIT_201s0010g02500.1	LHT	Phytozome 13
	VIT_201s0010g02520.1	LHT	Phytozome 13

VIT_201s0010g02640.1	LHT	Phytozome 13
VIT_201s0010g02660.1	LHT	Phytozome 13
VIT_201s0010g02700.1	LHT	Phytozome 13
VIT_201s0011g03180.1	LHT	Phytozome 13
VIT_201s0026g02500.1	ATLb	Phytozome 13
VIT_202s0025g01230.1	GAT	Phytozome 13
VIT_203s0038g02140.1	AUX	Phytozome 13
VIT_203s0038g02290.1	APP	Phytozome 13
VIT_203s0038g03530.1	LHT	Phytozome 13
VIT_203s0038g03540.1	LHT	Phytozome 13
VIT_203s0038g03900.1	ANT	Phytozome 13
VIT_204s0008g00230.1	APP	Phytozome 13
VIT_204s0008g00240.1	APP	Phytozome 13
VIT_204s0008g00250.1	APP	Phytozome 13
VIT_204s0008g00280.1	APP	Phytozome 13
VIT_205s0062g01130.1	ANT	Phytozome 13
VIT_205s0062g01150.1	ATLb	Phytozome 13
VIT_206s0009g01140.1	ATLb	Phytozome 13
VIT_206s0061g00570.1	ATLb	Phytozome 13
VIT_206s0061g01210.1	GAT	Phytozome 13
VIT_207s0031g02140.1	ANT	Phytozome 13
VIT_207s0031g02340.1	APP	Phytozome 13
VIT_207s0031g02440.1	APP	Phytozome 13
VIT_207s0141g00640.1	GAT	Phytozome 13
VIT_208s0007g02030.1	AUX	Phytozome 13
VIT_208s0007g05210.1	ATLb	Phytozome 13
VIT_209s0002g08410.1	ATLa	Phytozome 13
VIT_210s0116g01380.1	APP	Phytozome 13
VIT_213s0019g03220.1	PorT	Phytozome 13
VIT_213s0019g04660.1	ATLb	Phytozome 13
VIT_213s0067g00330.1	AUX	Phytozome 13
VIT_213s0067g02220.1	ATLa	Phytozome 13
VIT_213s0074g00540.1	GAT	Phytozome 13
VIT_213s0074g00570.1	GAT	Phytozome 13
VIT_214s0083g00740.1	ATLa	Phytozome 13
VIT_214s0108g00620.1	ATLb	Phytozome 13
VIT_214s0108g00630.1	ATLb	Phytozome 13
VIT_214s0108g00690.1	ATLb	Phytozome 13
VIT_214s0171g00400.1	LHT	Phytozome 13
VIT_218s0001g00320.1	LHT	Phytozome 13
VIT_218s0001g02020.1	APP	Phytozome 13
VIT_218s0001g03540.1	AUX	Phytozome 13
VIT_218s0001g08270.1	APP	Phytozome 13
VIT_218s0001g08570.1	ATLa	Phytozome 13

	VIT_218s0001g11300.1	LHT	Phytozome 13
	VIT_218s0041g01240.1	APP	Phytozome 13
	VIT_218s0041g01250.1	APP	Phytozome 13
	VIT_218s0041g01270.1	APP	Phytozome 13
	VIT_219s0015g01290.1	APP	Phytozome 13
	VIT_219s0015g01300.1	APP	Phytozome 13
	VIT_219s0027g01860.1	ATLb	Phytozome 13
	VIT_219s0027g01870.1	ATLb	Phytozome 13
	VIT_219s0027g01880.1	ATLb	Phytozome 13
	VIT_219s0027g01890.1	ATLb	Phytozome 13
	Zm00001d001984_P001	APP	Phytozome 13
	Zm00001d002176_P001	LHT	Phytozome 13
	Zm00001d002673_P001	LHT	Phytozome 13
	Zm00001d003343_P001	APP	Phytozome 13
	Zm00001d003403_P001	LHT	Phytozome 13
	Zm00001d003422_P001	ATLb	Phytozome 13
	Zm00001d004340_P001	ATLb	Phytozome 13
	Zm00001d004633_P001	ATLb	Phytozome 13
	Zm00001d004705_P001	APP	Phytozome 13
	Zm00001d010231_P001	APP	Phytozome 13
	Zm00001d012229_P001	APP	Phytozome 13
	Zm00001d012231_P001	APP	Phytozome 13
	Zm00001d012485_P001	ATLa	Phytozome 13
	Zm00001d015658_P001	ATLa	Phytozome 13
	Zm00001d017557_P001	ANT	Phytozome 13
	Zm00001d018336_P001	ATLb	Phytozome 13
Zea mays	Zm00001d018340_P001	ATLb	Phytozome 13
	Zm00001d018598_P001	PorT	Phytozome 13
	Zm00001d018751_P001	APP	Phytozome 13
	Zm00001d019225_P001	ANT	Phytozome 13
	Zm00001d023592_P001	APP	Phytozome 13
	Zm00001d023593_P001	APP	Phytozome 13
	Zm00001d023596_P001	APP	Phytozome 13
	Zm00001d023603_P001	APP	Phytozome 13
	Zm00001d024204_P001	LHT	Phytozome 13
	Zm00001d025665_P001	APP	Phytozome 13
	Zm00001d026121_P001	ANT	Phytozome 13
	Zm00001d026131_P001	LHT	Phytozome 13
	Zm00001d028401_P001	AUX	Phytozome 13
	Zm00001d030310_P001	AUX	Phytozome 13
	Zm00001d030751_P001	ATLb	Phytozome 13
	Zm00001d031922_P001	LHT	Phytozome 13
	Zm00001d035157_P001	LHT	Phytozome 13

	Zm00001d035161_P001	LHT	Phytozome 13
	Zm00001d035162_P001	LHT	Phytozome 13
	Zm00001d035163_P001	LHT	Phytozome 13
	Zm00001d035166_P001	LHT	Phytozome 13
	Zm00001d035243_P001	APP	Phytozome 13
	Zm00001d036984_P001	APP	Phytozome 13
	Zm00001d037057_P001	ATLb	Phytozome 13
	Zm00001d037229_P001	ATLb	Phytozome 13
	Zm00001d037789_P001	LHT	Phytozome 13
	Zm00001d041004_P001	LHT	Phytozome 13
	Zm00001d041534_P001	ATLb	Phytozome 13
	Zm00001d041836_P001	APP	Phytozome 13
	Zm00001d041842_P001	APP	Phytozome 13
	Zm00001d042438_P001	PorT	Phytozome 13
	Zm00001d042619_P001	APP	Phytozome 13
	Zm00001d042636_P001	APP	Phytozome 13
	Zm00001d042801_P001	GAT	Phytozome 13
	Zm00001d044192_P001	GAT	Phytozome 13
	Zm00001d044387_P001	ATLb	Phytozome 13
	Zm00001d045740_P001	ATLa	Phytozome 13
	Zm00001d046235_P002	APP	Phytozome 13
	Zm00001d046263_P001	APP	Phytozome 13
	Zm00001d046586_P001	ATLa	Phytozome 13
	Zm00001d046661_P001	ATLa	Phytozome 13
	Zm00001d048577_P001	ATLb	Phytozome 13
	Zm00001d049640_P002	LHT	Phytozome 13
	Zm00001d050211_P001	APP	Phytozome 13
	Zm00001d051703_P001	ATLa	Phytozome 13
	Zm00001d052063_P001	ATLb	Phytozome 13
	Zm00001d053004_P001	AUX	Phytozome 13
	Zm00001d053143_P001	APP	Phytozome 13
