# Supplementary material for: Identification, Phylogenetic and Expression Analyses of the AAAP Gene Family in Liriodendron chinense Reveal Their Putative Functions in Response to Organ and Multiple Abiotic Stresses
Source: Int J Mol Sci. 2022 Apr 26;23(9):4765. doi: 10.3390/ijms23094765 (PMC9100865; doi:10.3390/ijms23094765)
Supplement: Supplementary file 1 [file ijms-23-04765-s001.zip › Table S2.pdf]

Supplementary Table S2 The 20 motifs in LcAAAP proteins

Motif number	Motif Sequence	Motif Logo	Width
Motif 1	MKKAVRISIAITTAFYFSVGCFGYLAF GBST	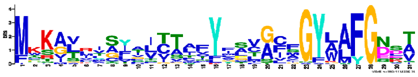	31
Motif 2	FYEPFWLVDIANLAIVIHLVGAYQVFC QP	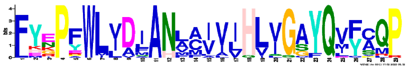	29
Motif 3	SGSEKIWRVFQALGBIAFAYAGSIVLIE IQDTLKSPPPENK	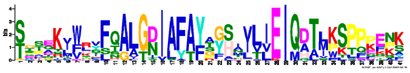	41
Motif 4	AVFHLVTAIVGAGVLSLPYALKQLGW GPG	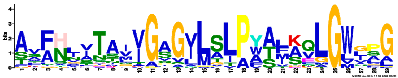	29
Motif 5	LLSDCYRSPDPVTGKRNYTYMDAVR ANLG	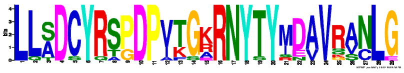	29
Motif 6	VIAMMFPFFGDLFGFLGAFAF	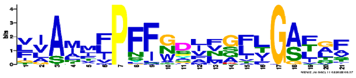	21
Motif 7	LSQIPNFHSJRWJSJVAAVMS	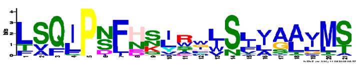	21
Motif 8	WPLTVYFPVEMYJAQKKIKRW	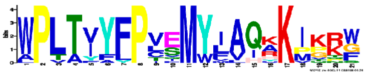	21
Motif 9	YTITASISMMAIKRSNCFHKNGHKAP CSA	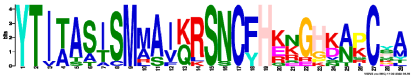	29
Motif 10	HFAVLEKWLGKHWPNSRFFTN	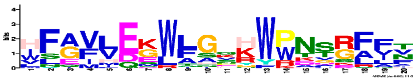	21
Motif 11	YSSVLNDAVRVSYALHLMLVFPJLFFS LRJNLDGLJFPKSRPLVSDNTRF	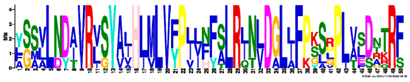	50
Motif 12	ETPRWLPDJSGKVSFLDLFTAVPVJVT AYTCHFNVHPIQNE	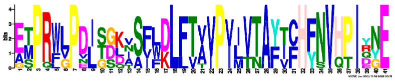	41
Motif 13	LQICIVINNMGVLIIYLIIIGDVLSGSSS	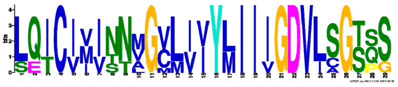	29
Motif 14	CTFLLFGSVIQLIACASNIYYINDNLD KRTWTYIFGACCAT	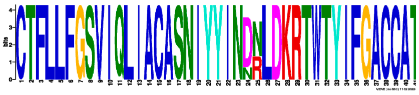	41
Motif 15	YLLATLYVLTLTLPSASSVYWAFGDM LLNHSNAFALLPKSGFRDMAVILM	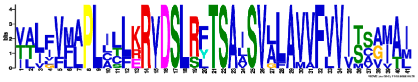	41
Motif 16	SGRISSYPDVGQHAFGRKGRJ	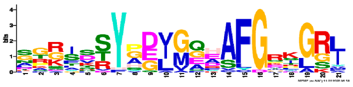	21
Motif 17	VCIGFIFPAAIVLRDVQGISTKRDKILA A	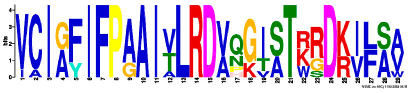	29
Motif 18	YLLATLYVLTLTLPSASSVYWAFGDM LLNHSNAFALLPKSGFRDMAVILM	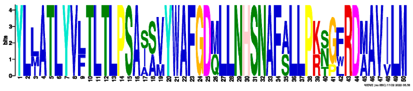	50

Motif 19	LFQLFYGJMGSWTAYLISVLYVEYRTRKEREKVDFRNHVIQ	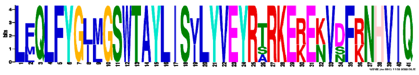	41
Motif 20	HQFITFGFACTPLYFVWEKAIGMHETKSICKRAJARLPVVI	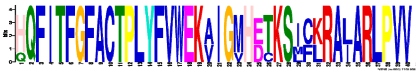	41
