# Supplementary figures and images for: Identification, Phylogenetic and Expression Analyses of the AAAP Gene Family in Liriodendron chinense Reveal Their Putative Functions in Response to Organ and Multiple Abiotic Stresses
Source: Int J Mol Sci. 2022 Apr 26;23(9):4765. doi: 10.3390/ijms23094765 (PMC9100865; doi:10.3390/ijms23094765)

AUX

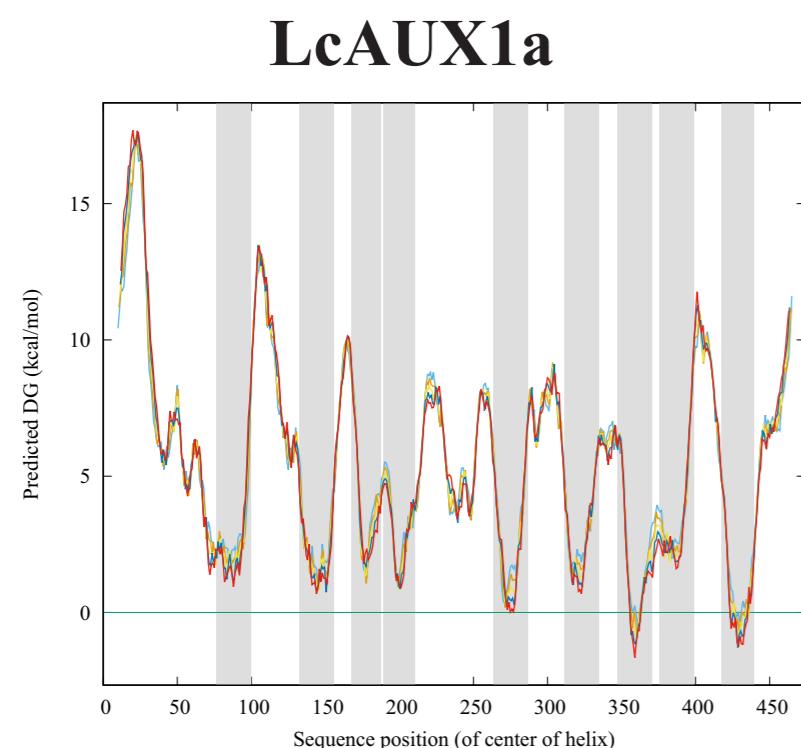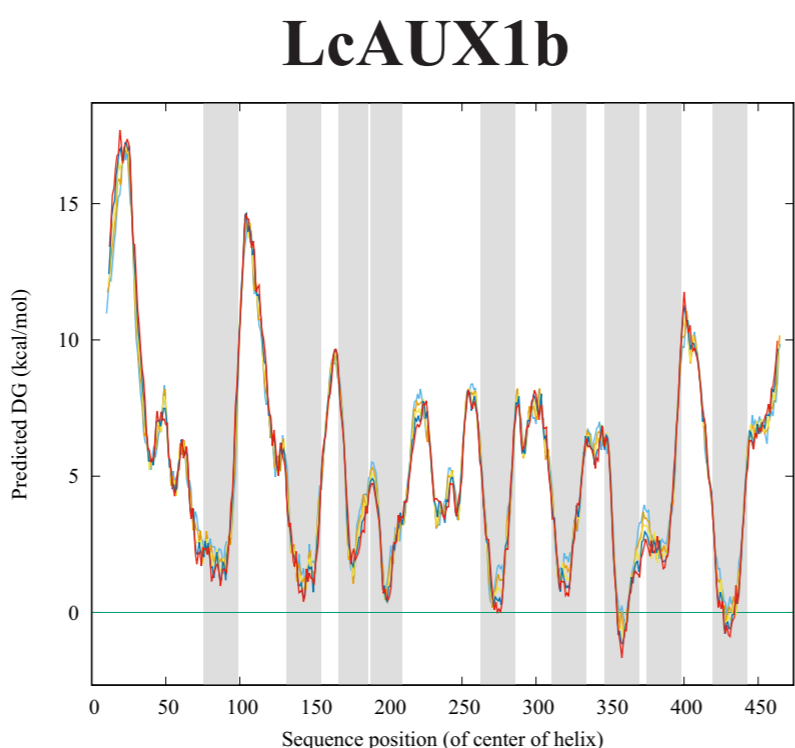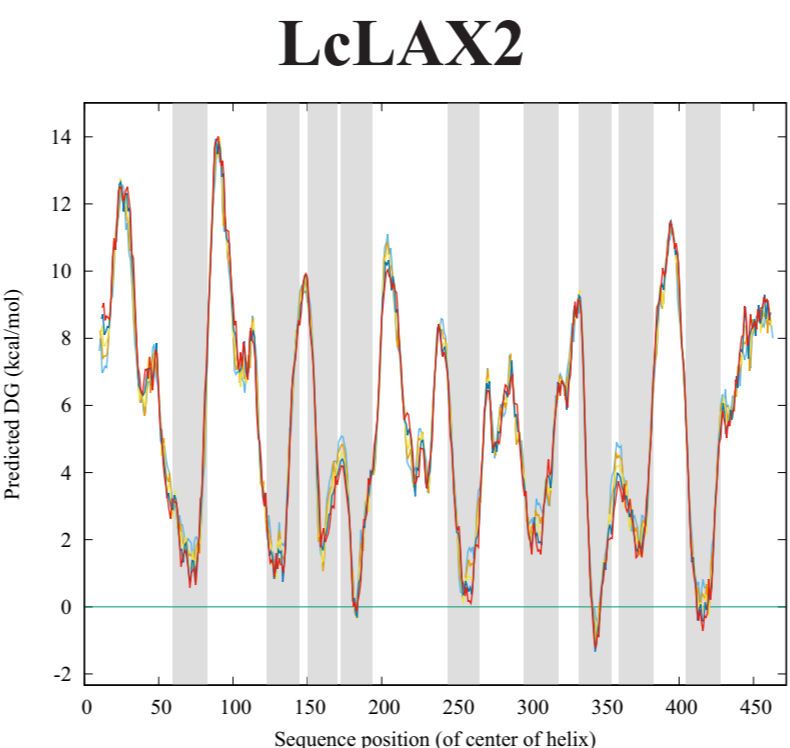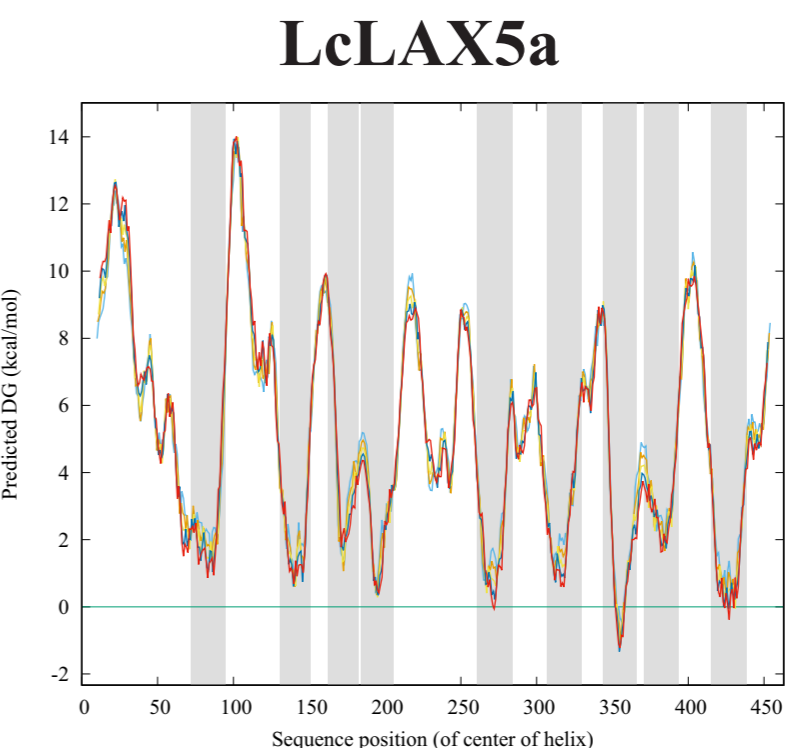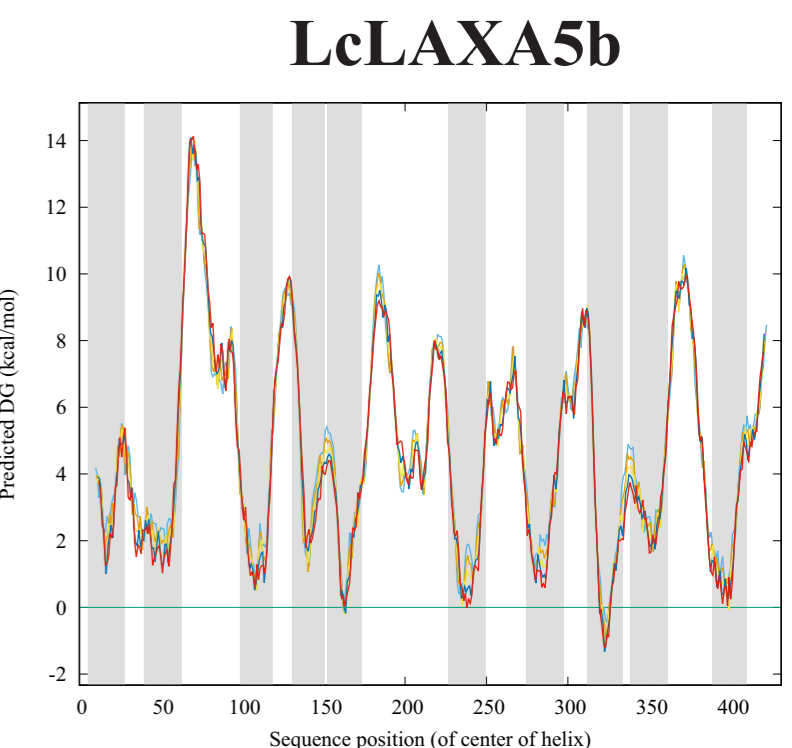

ANT

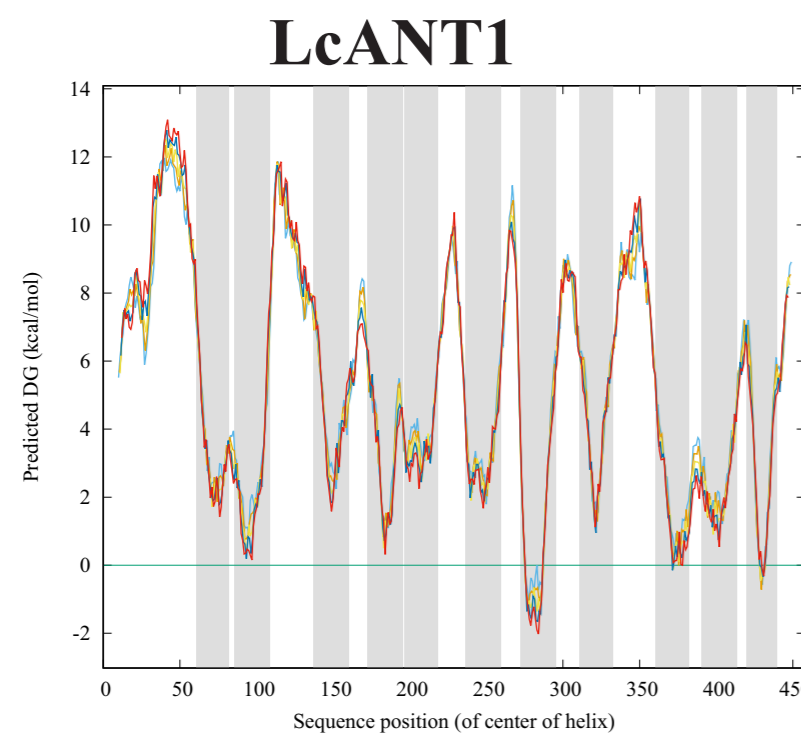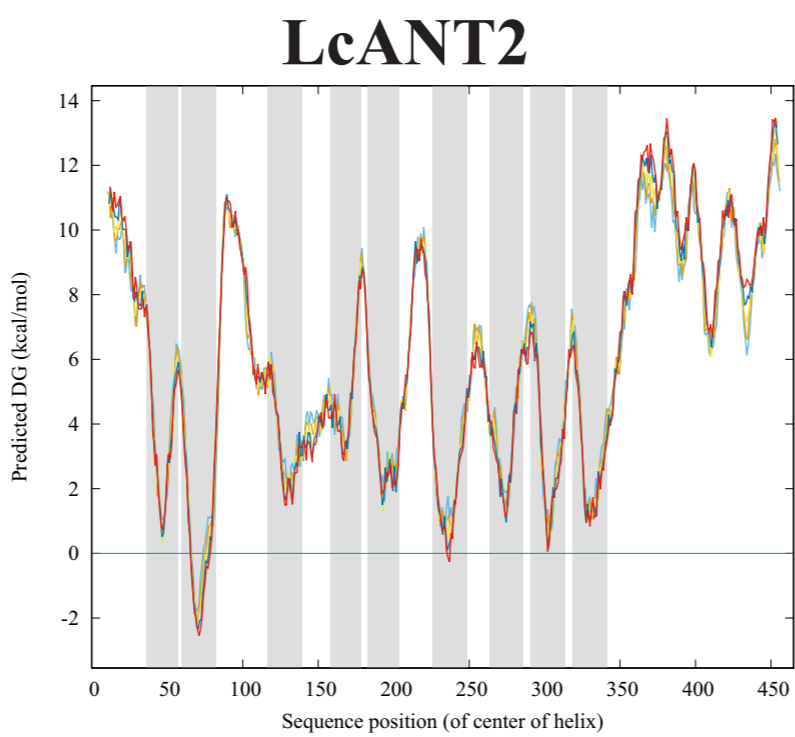

ATLa

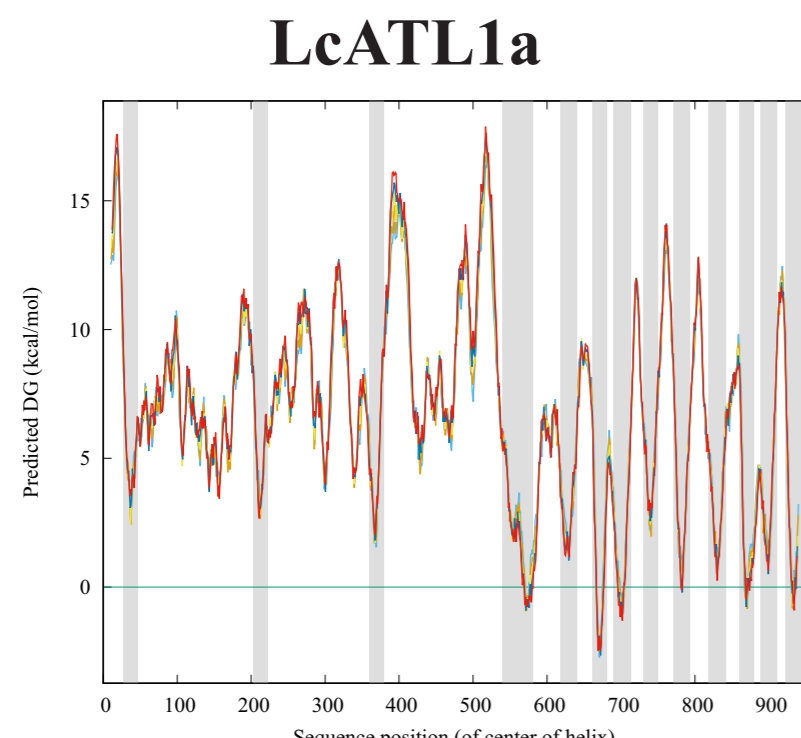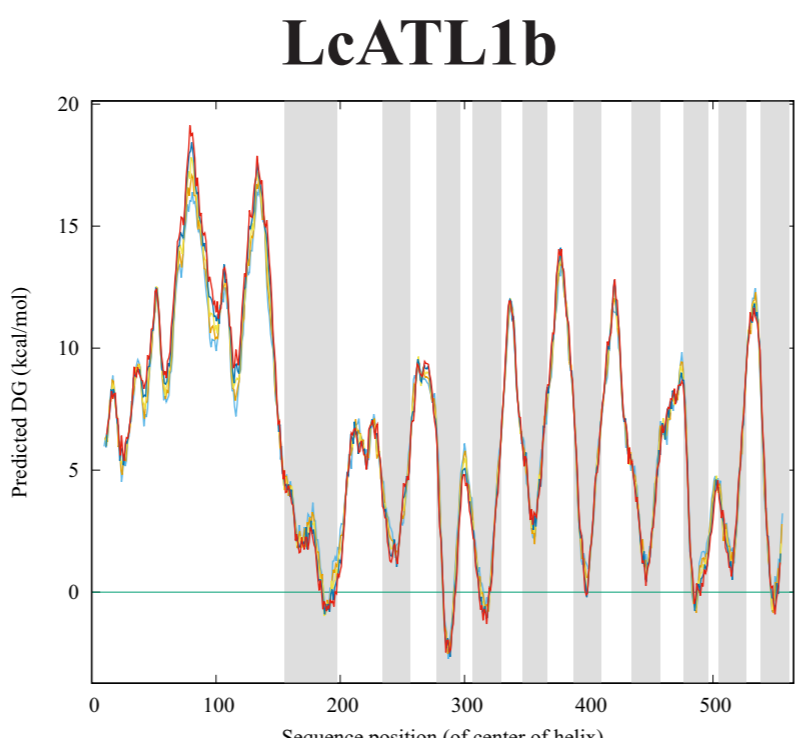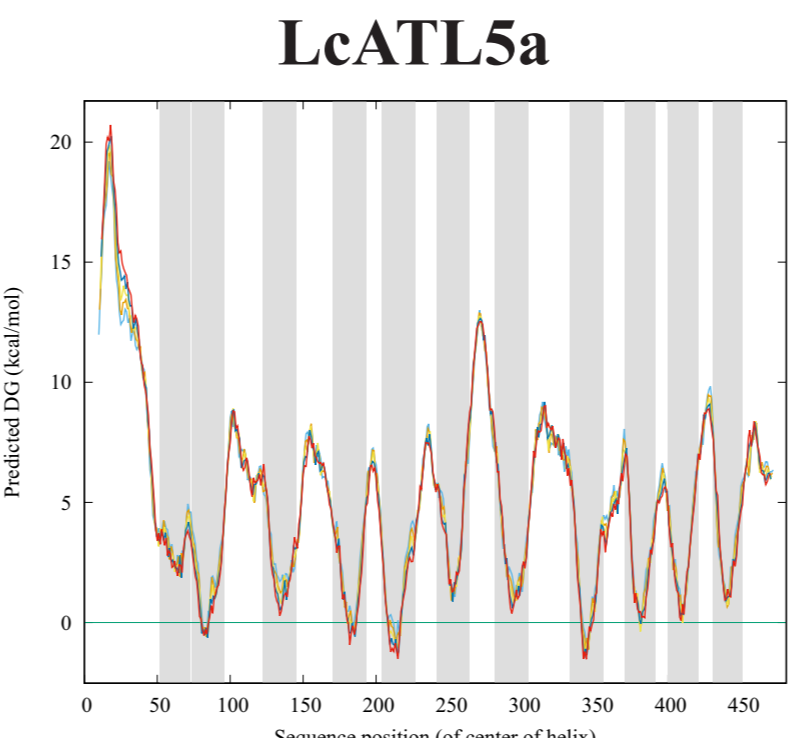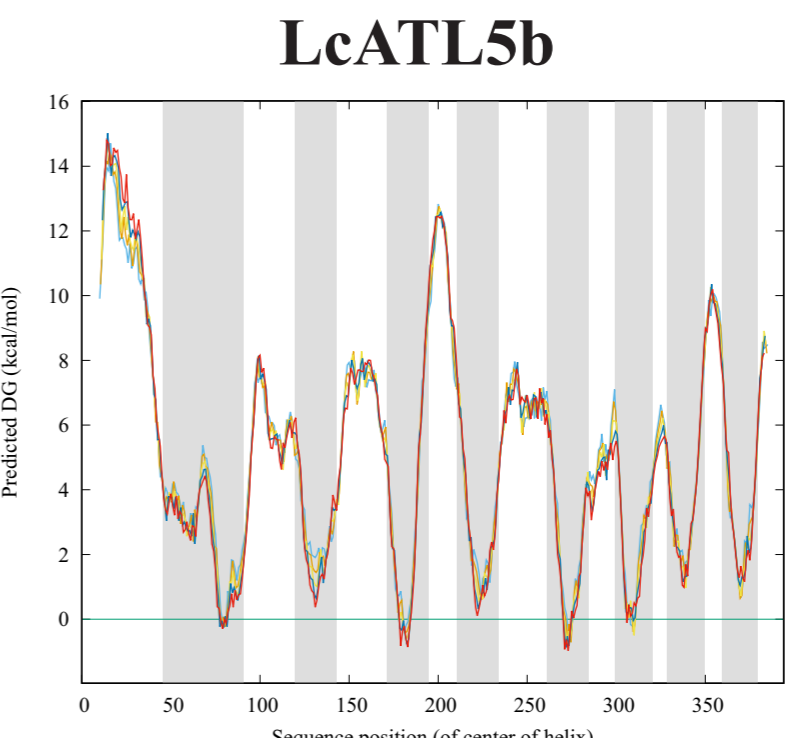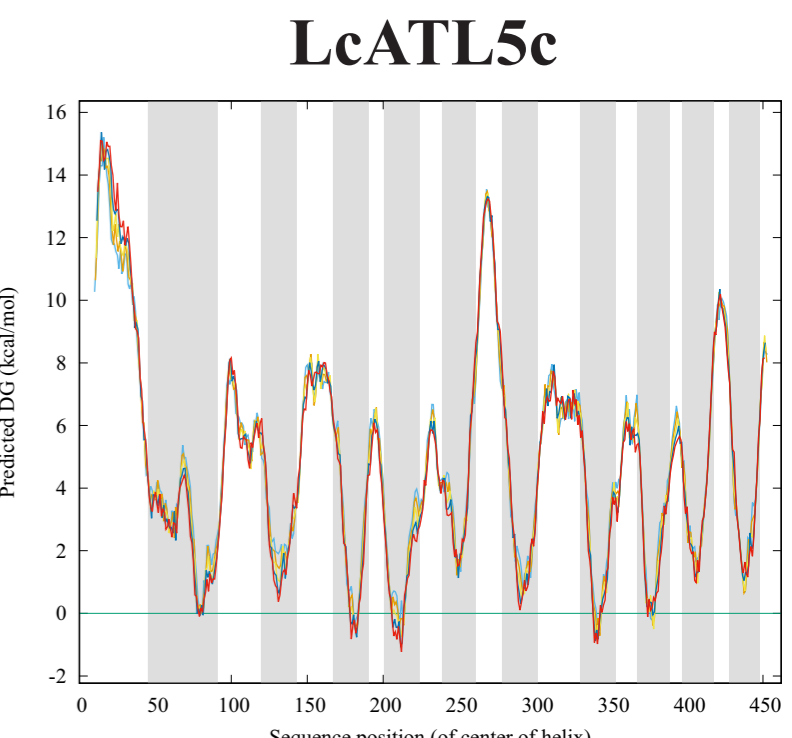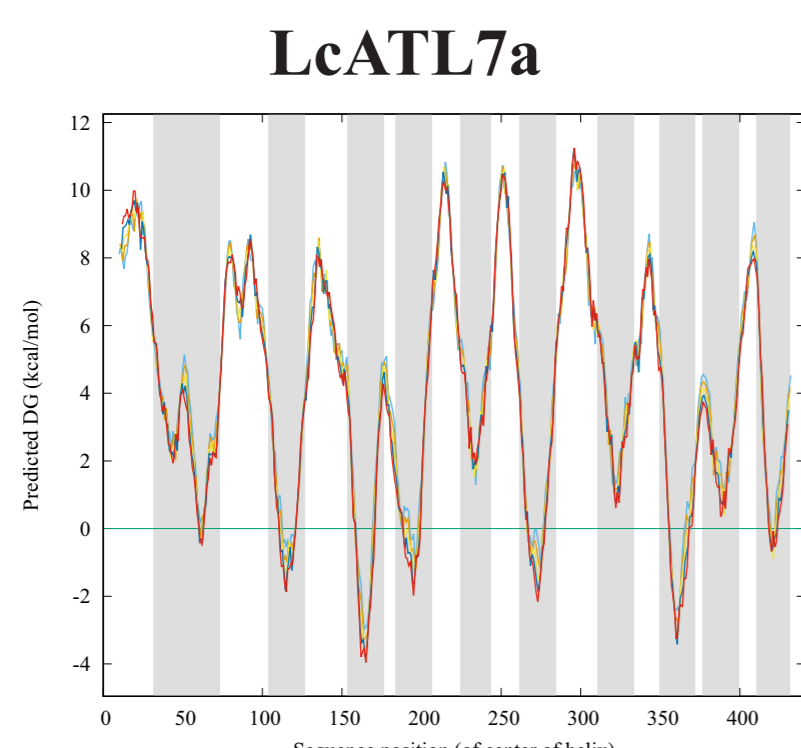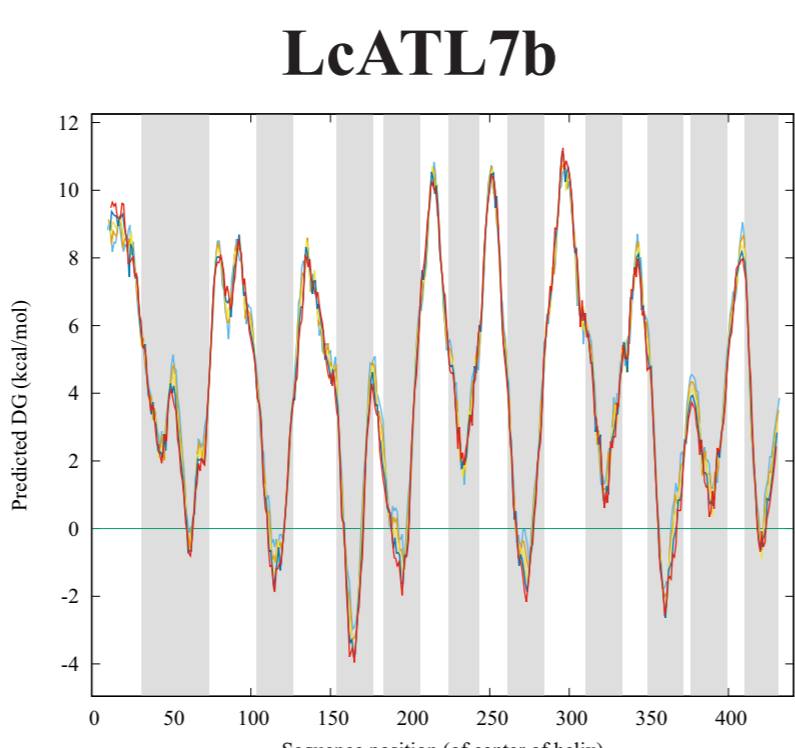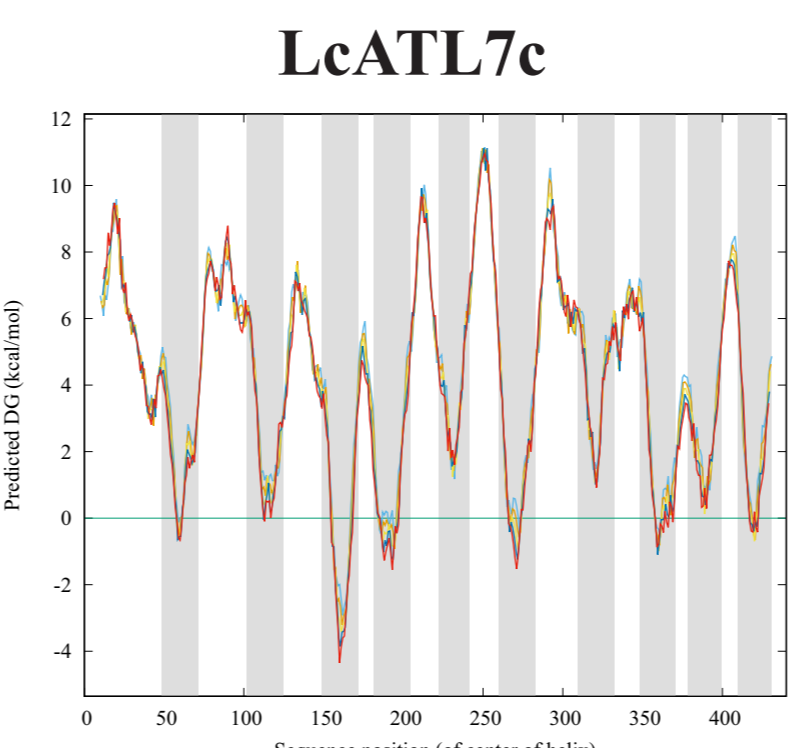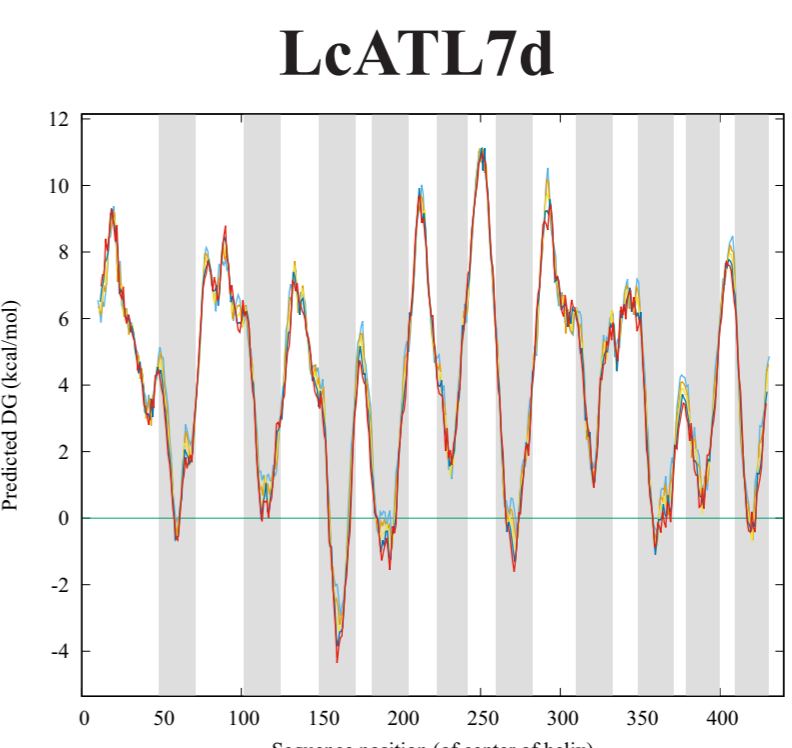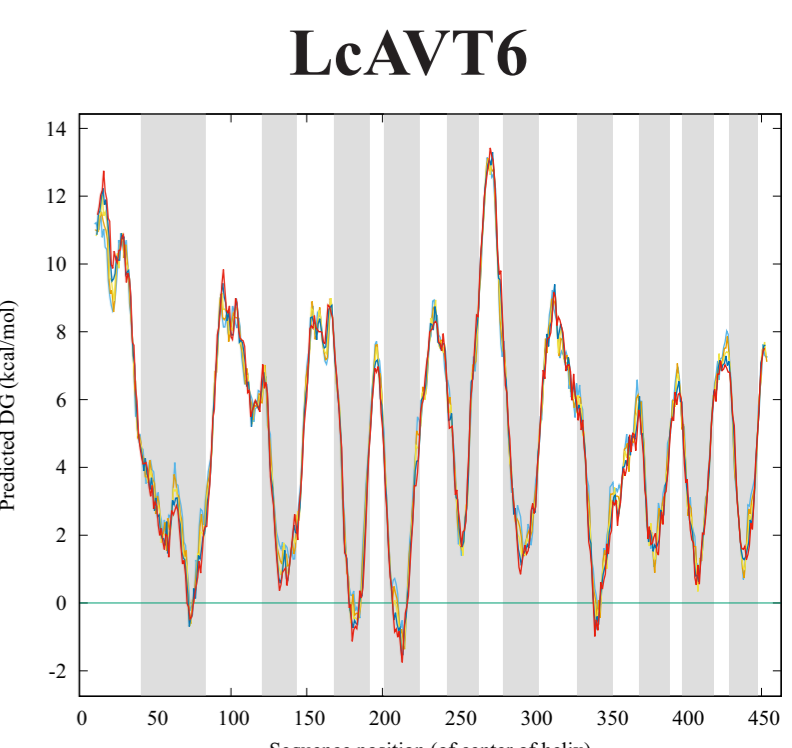

ATLb

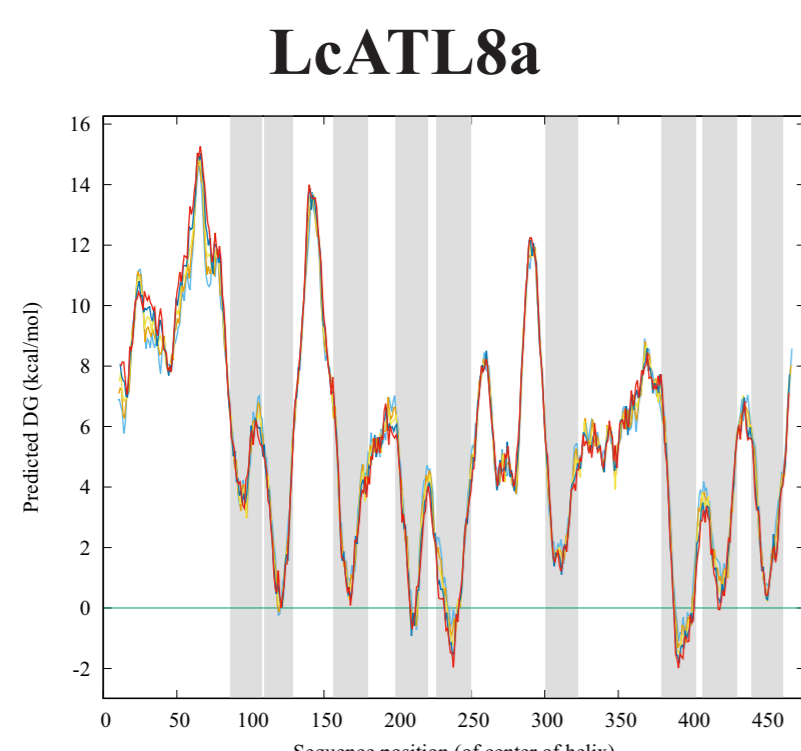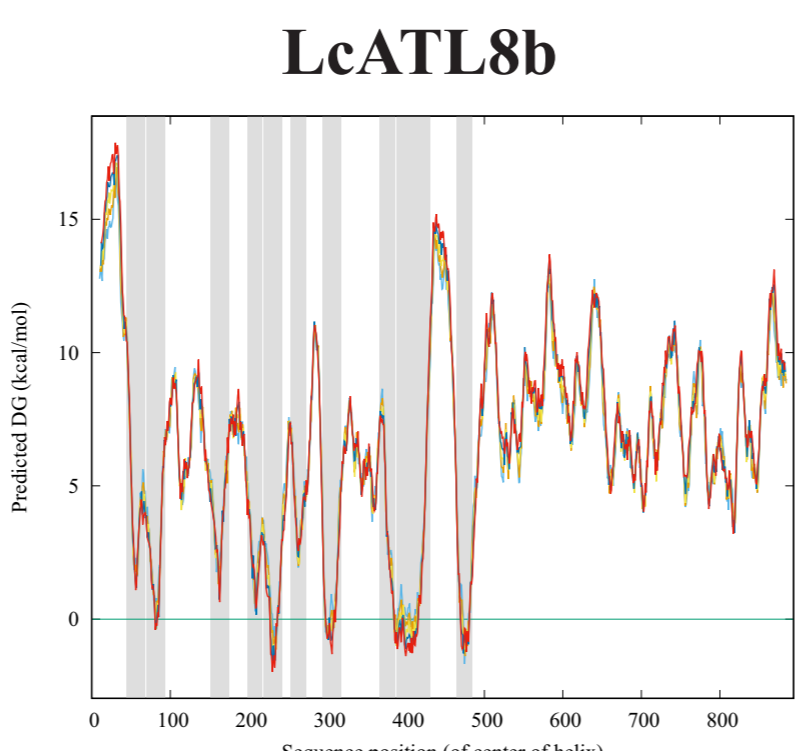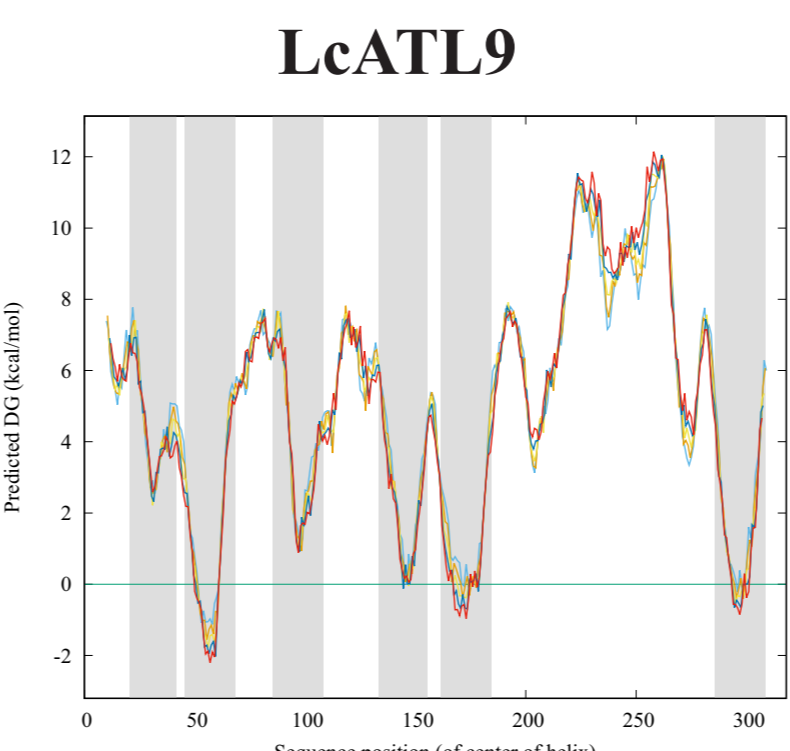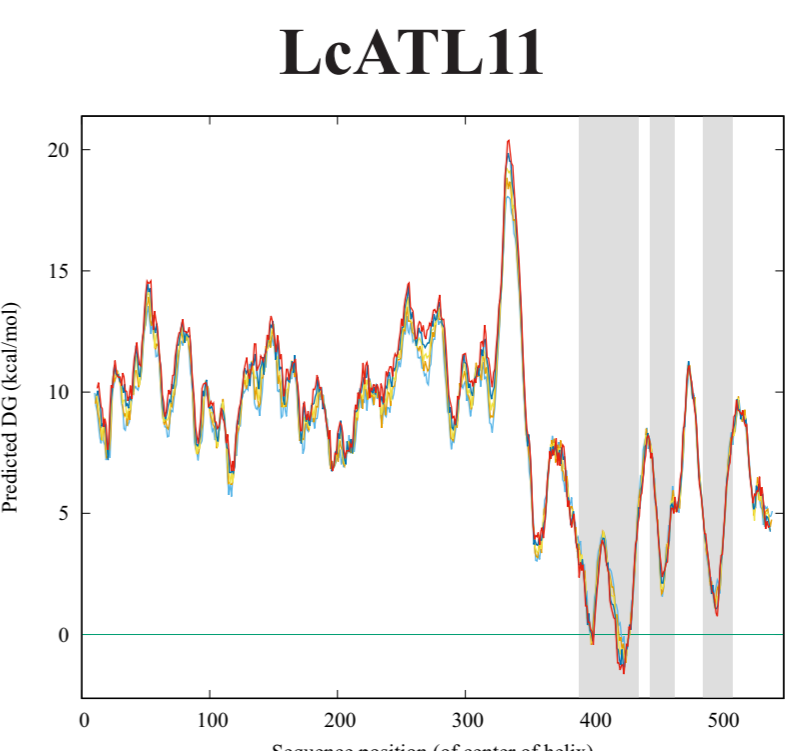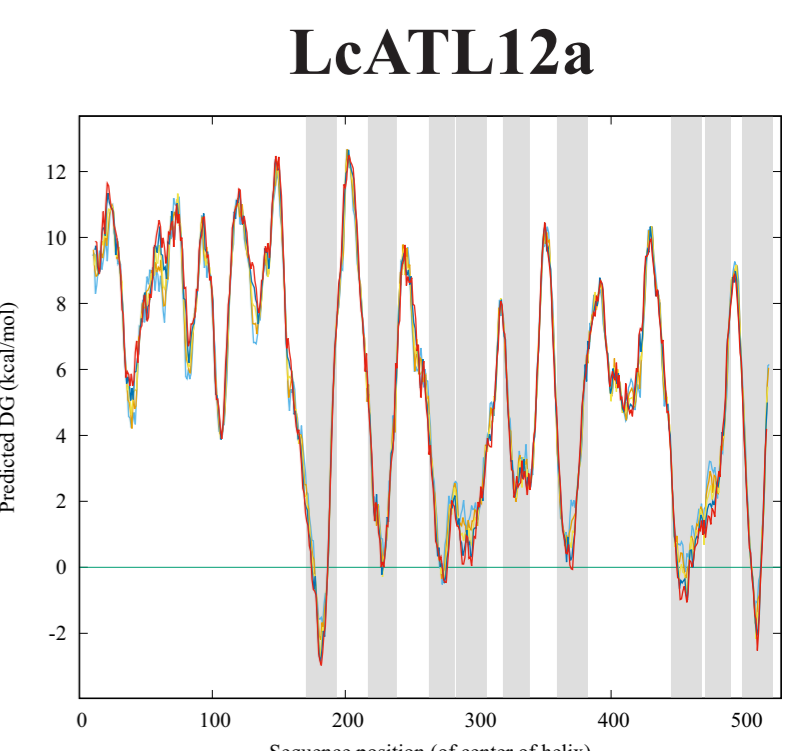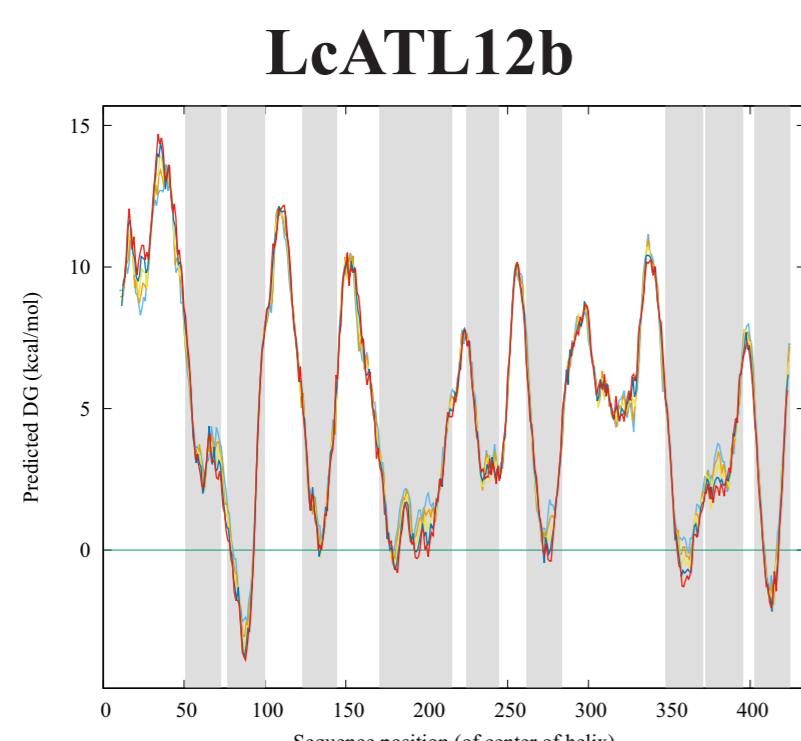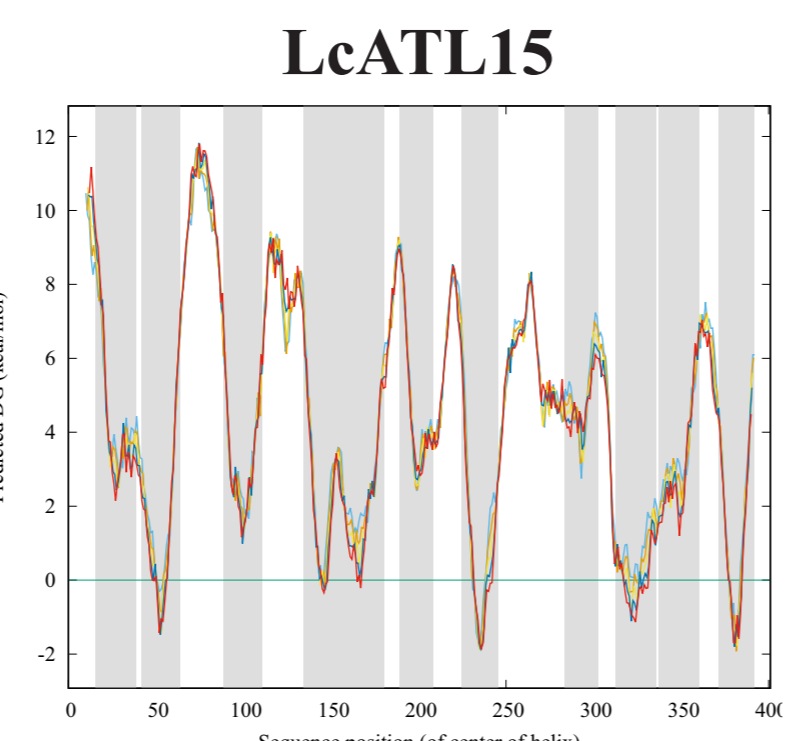

LHT

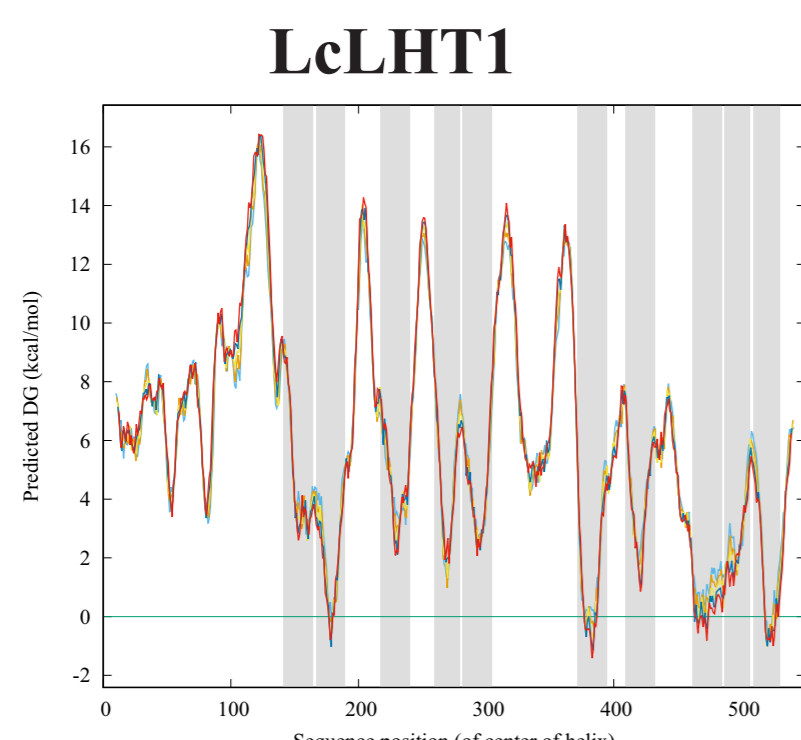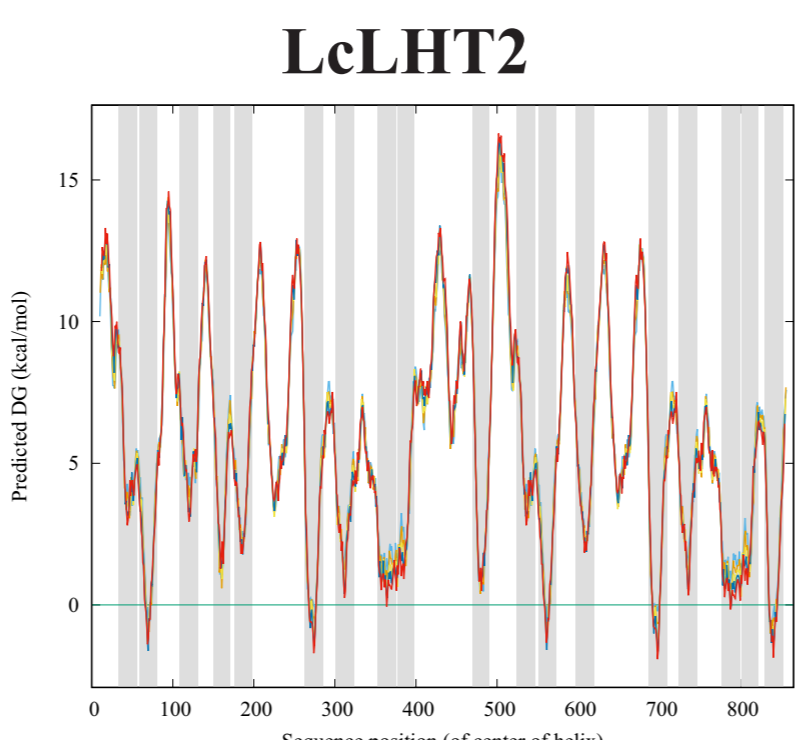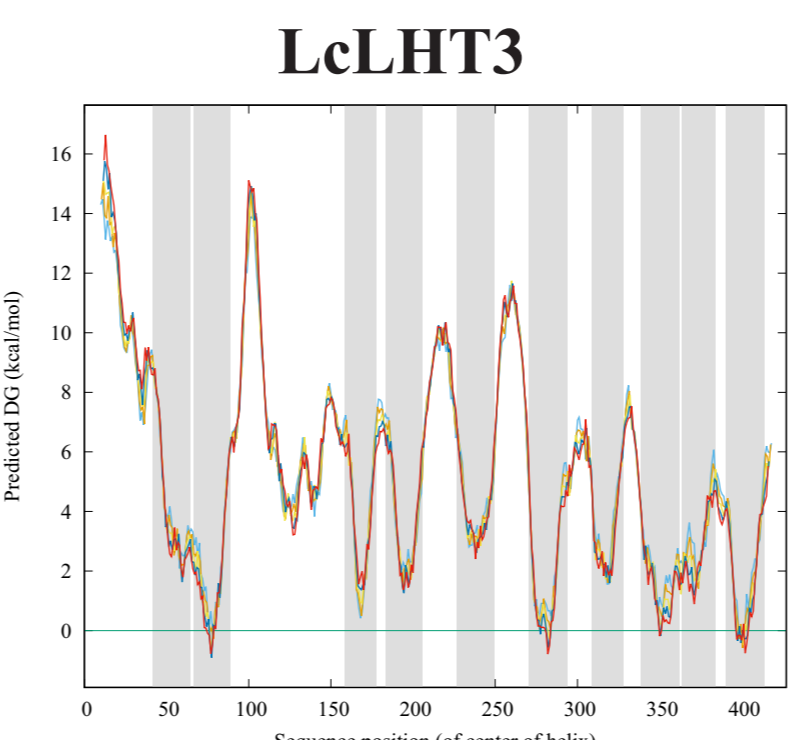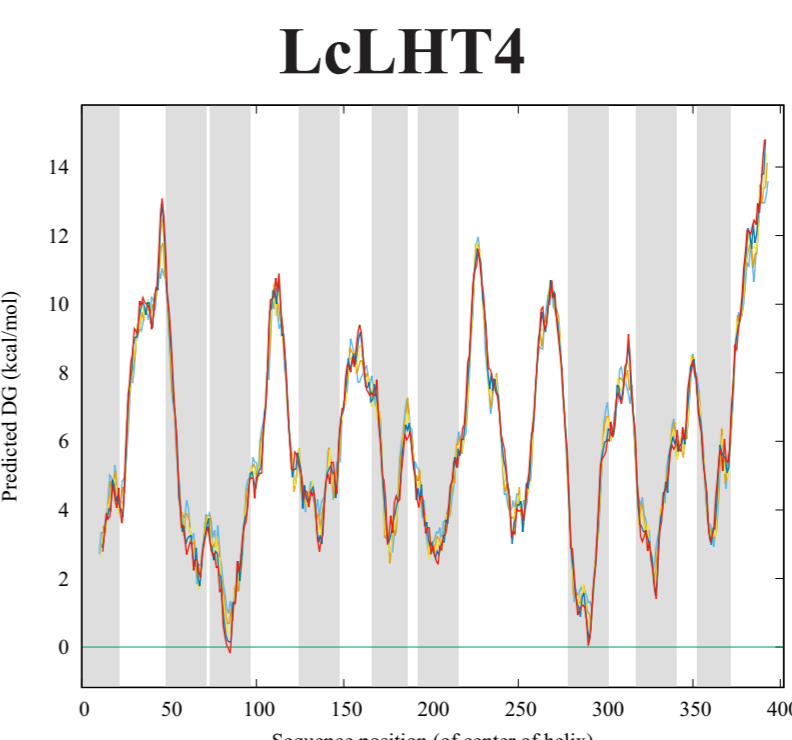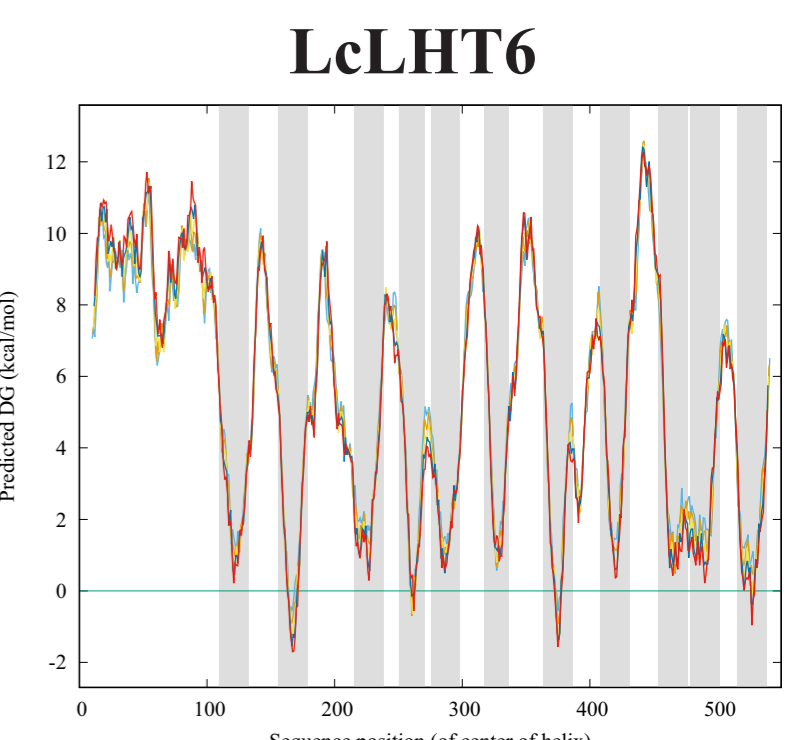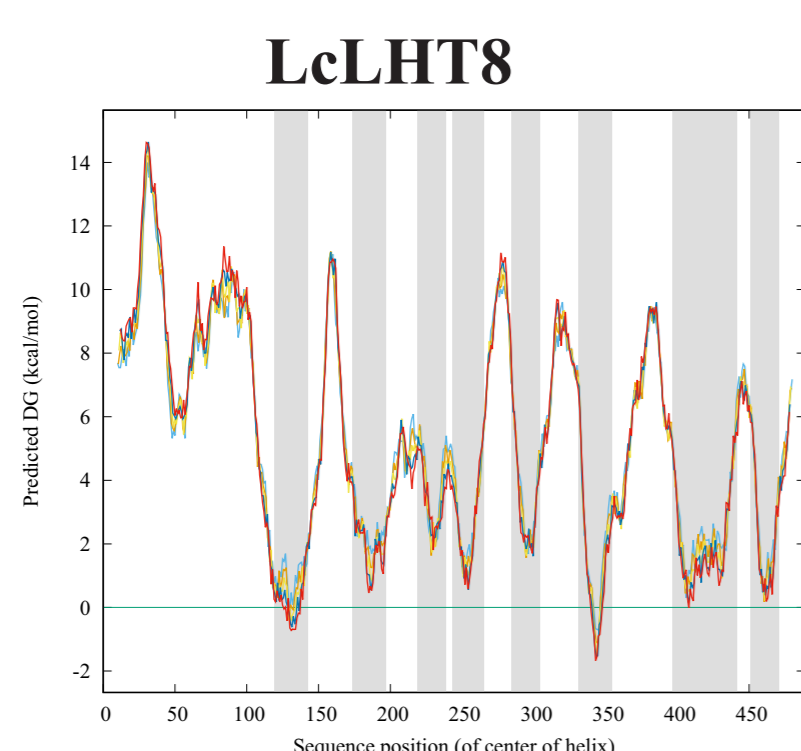

GAT

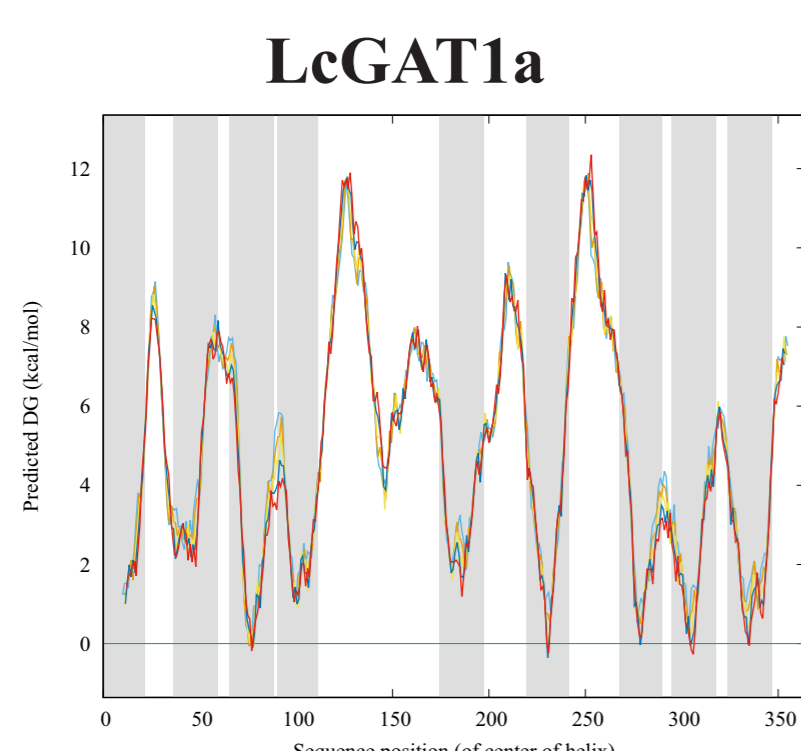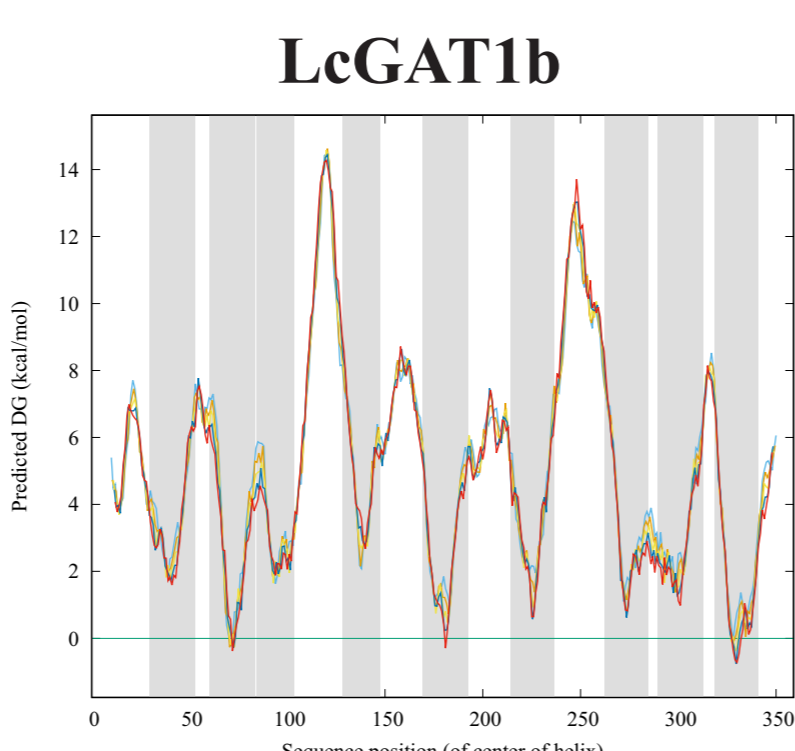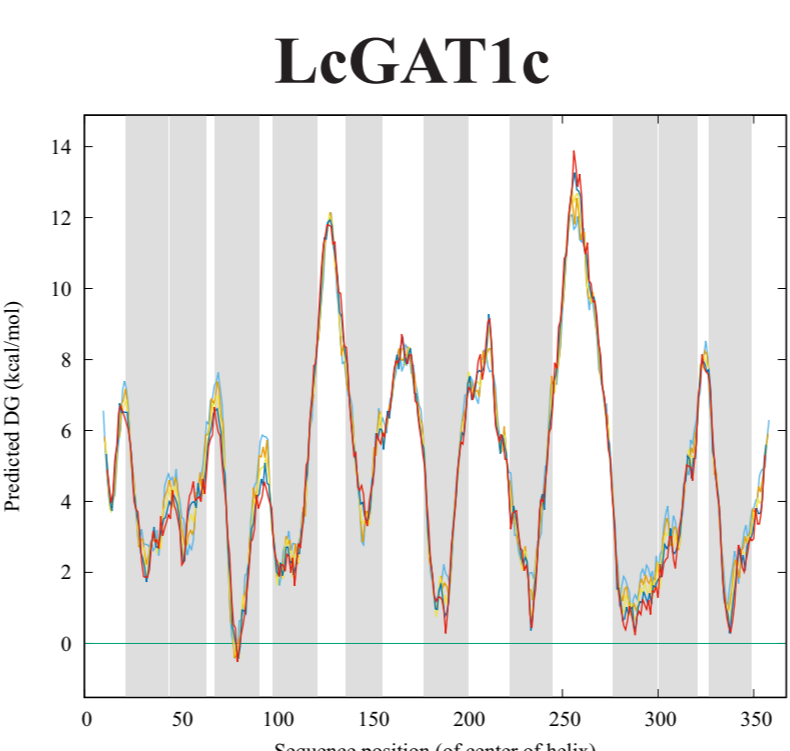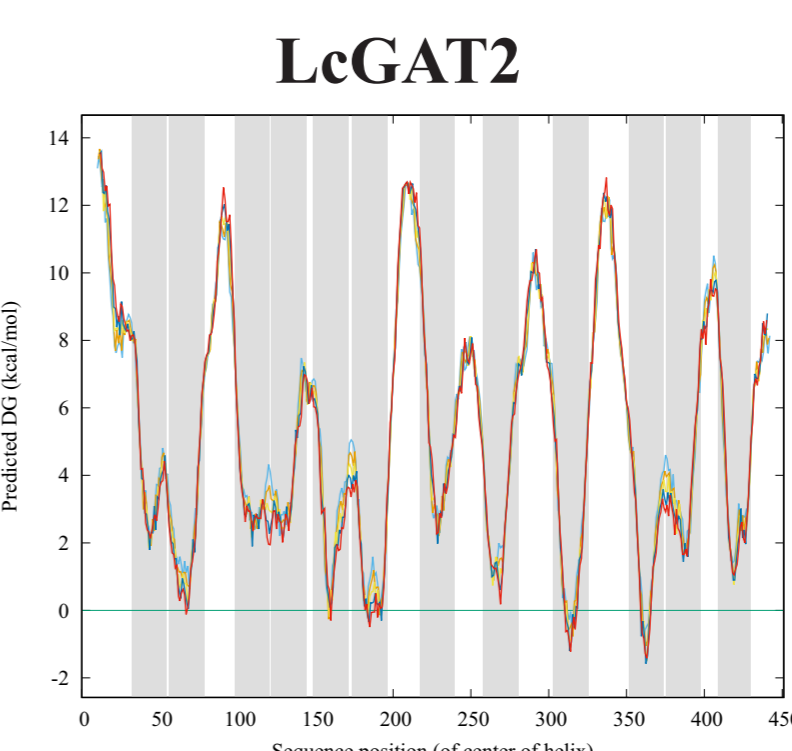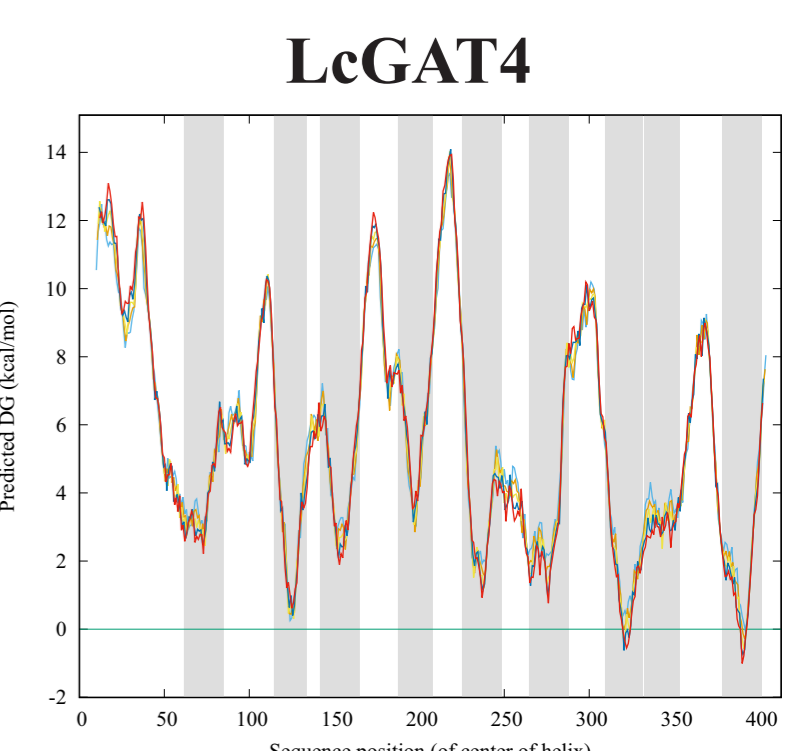

AAP

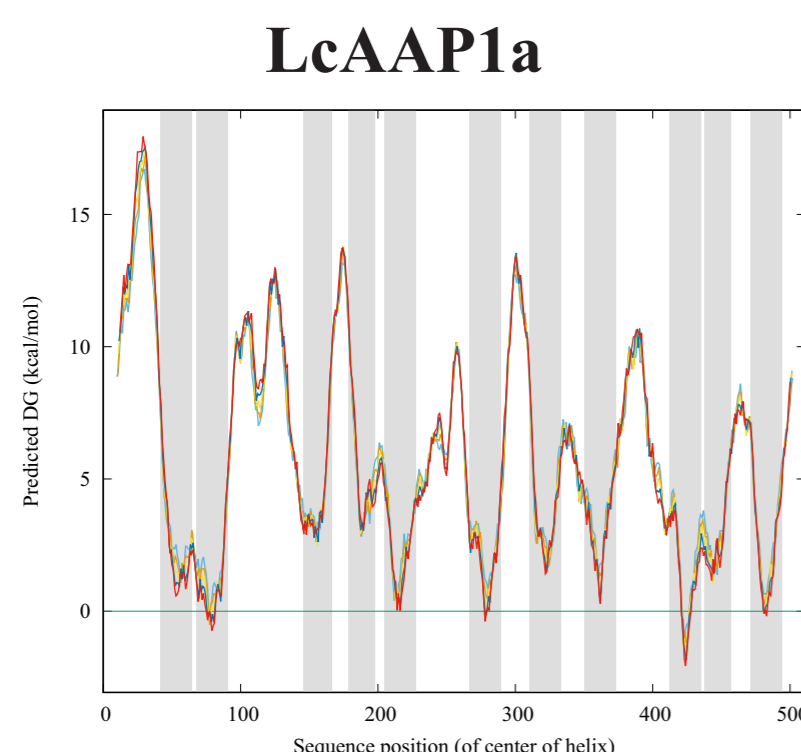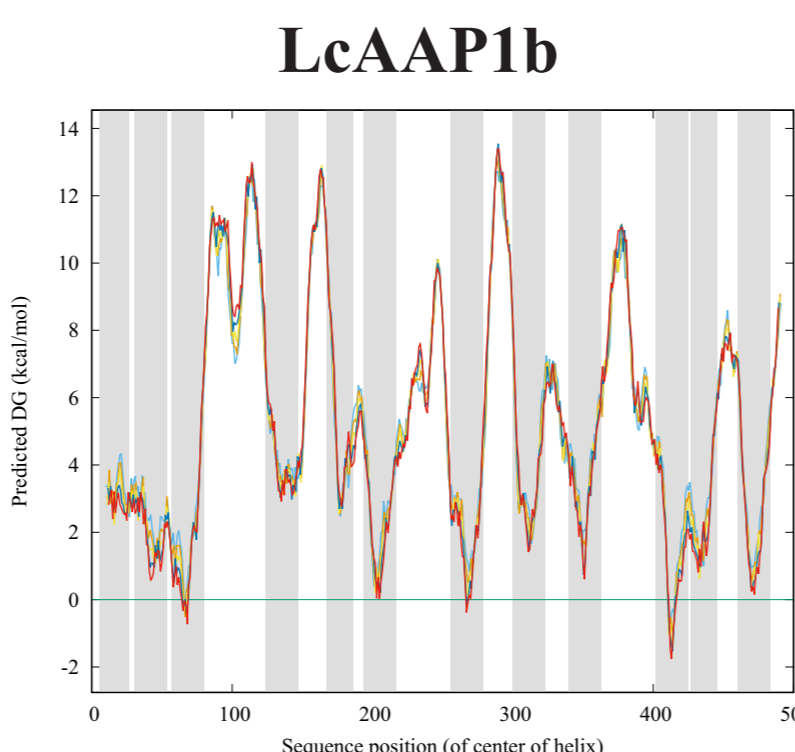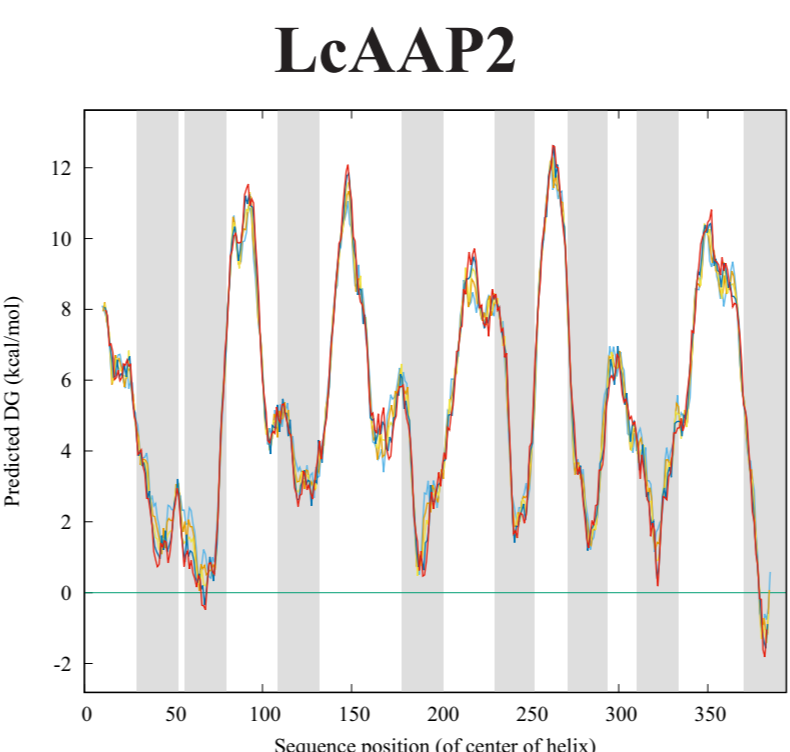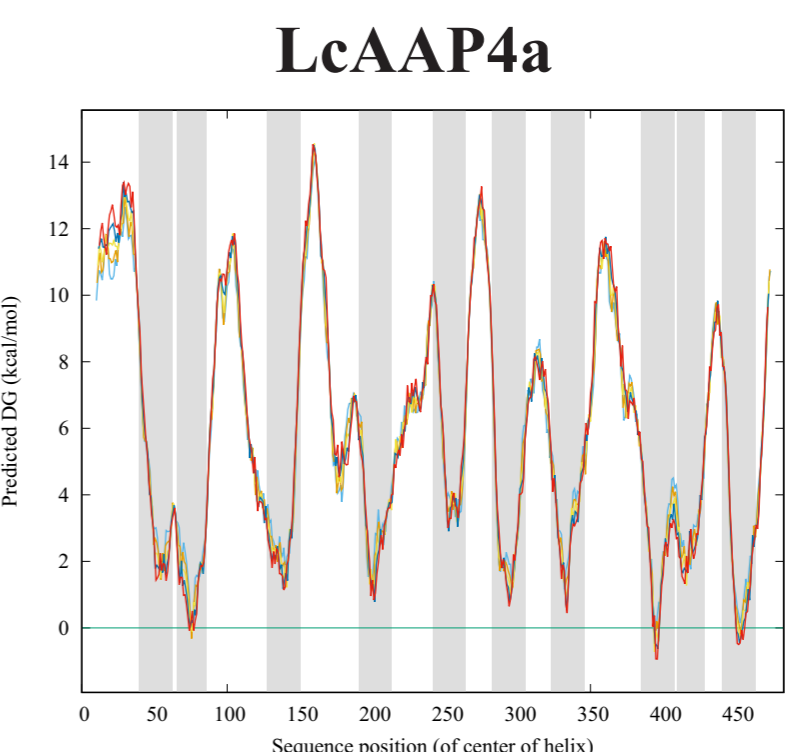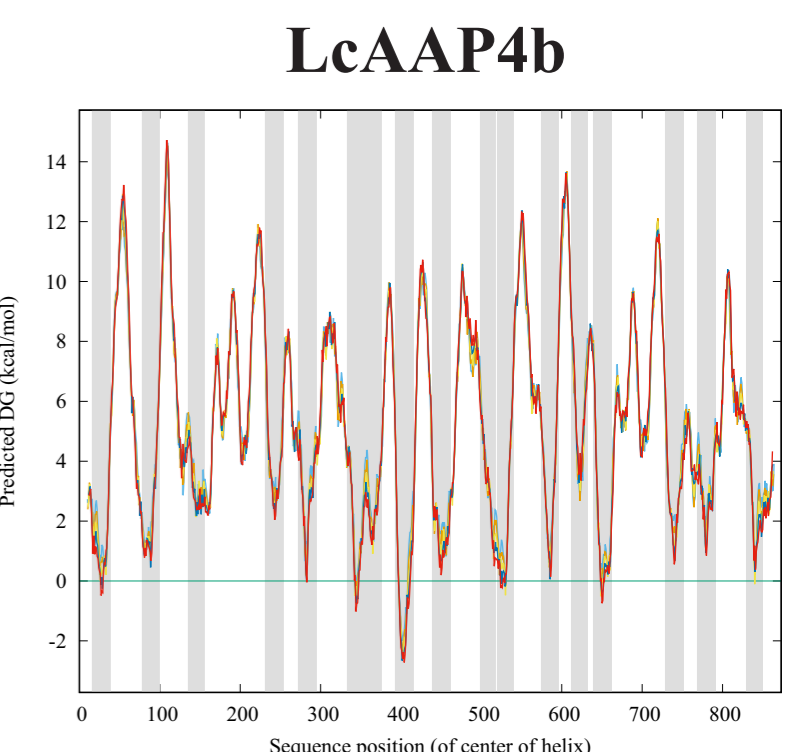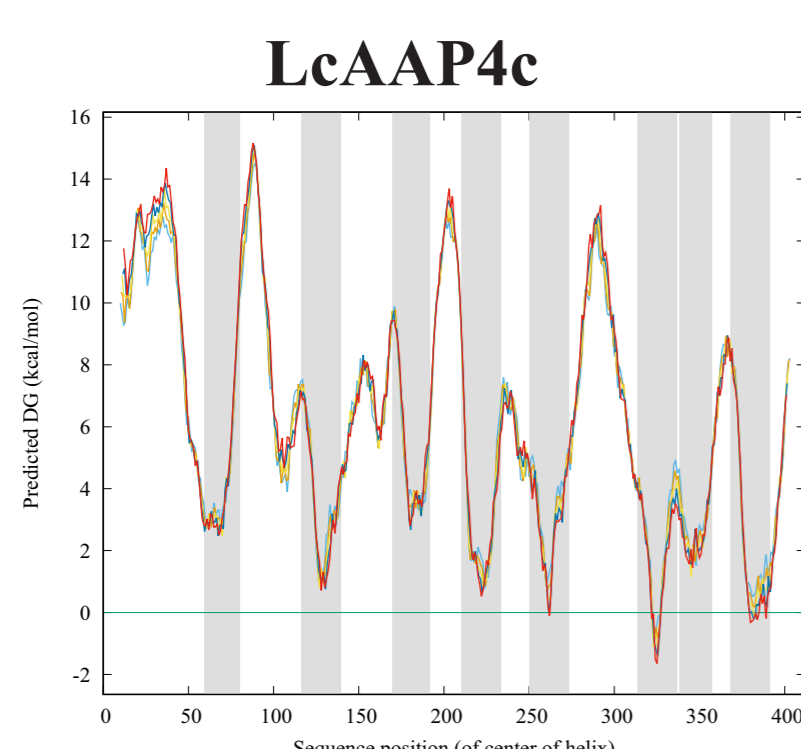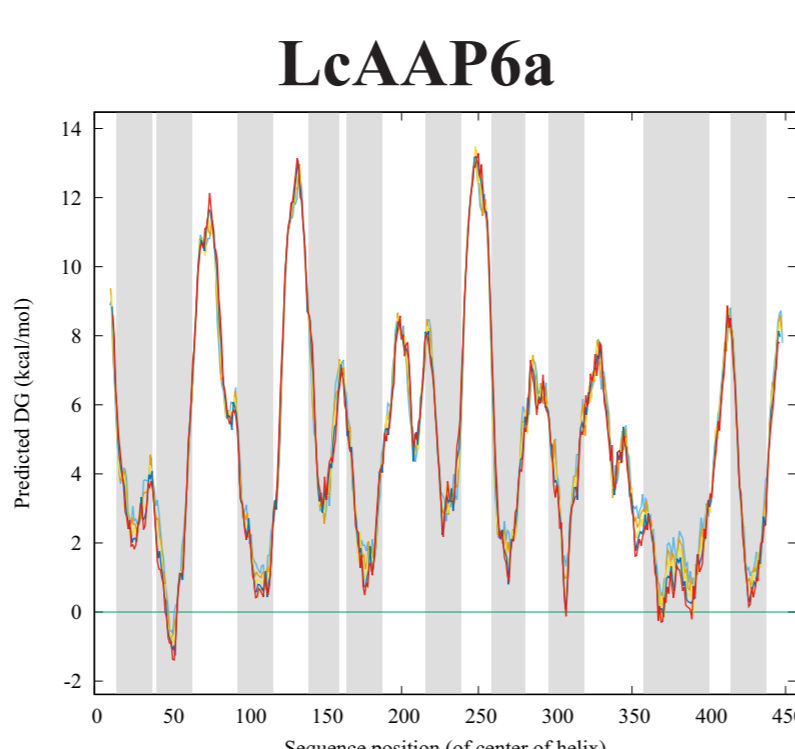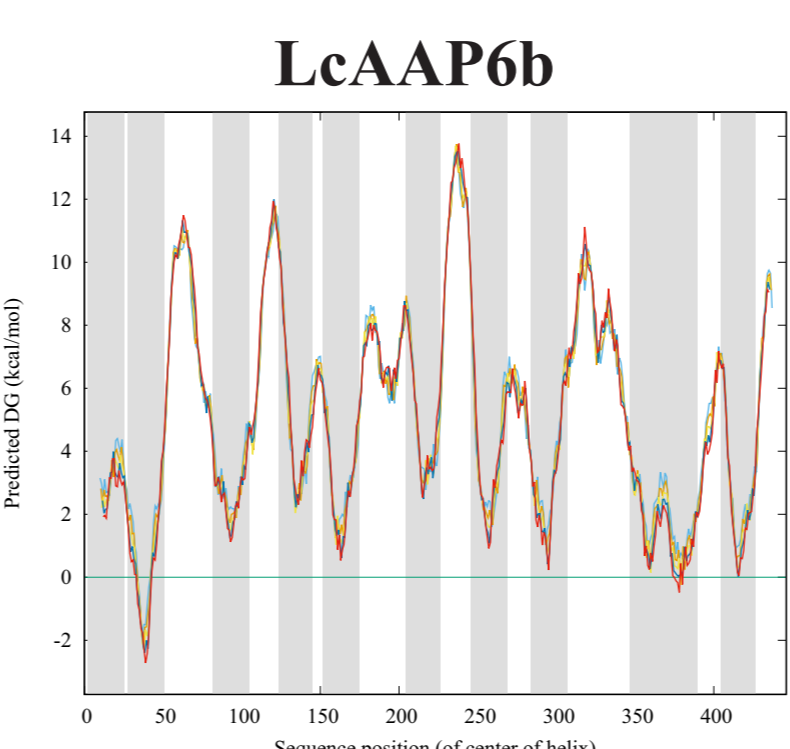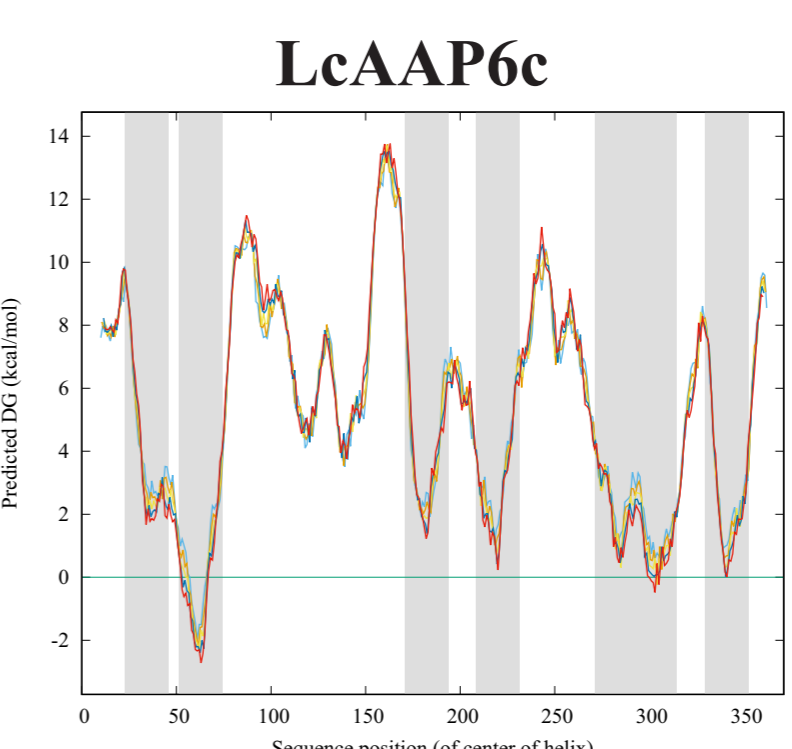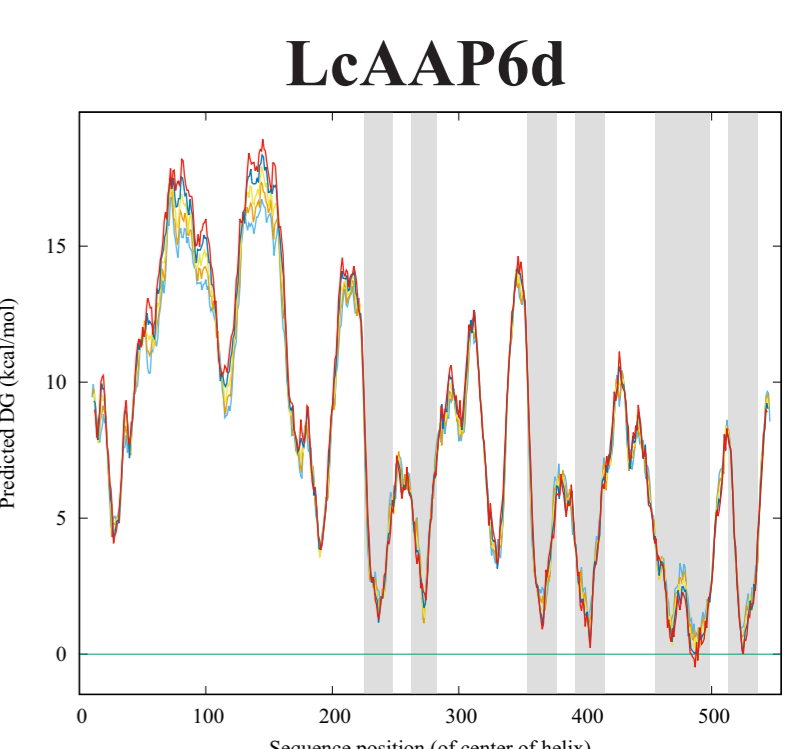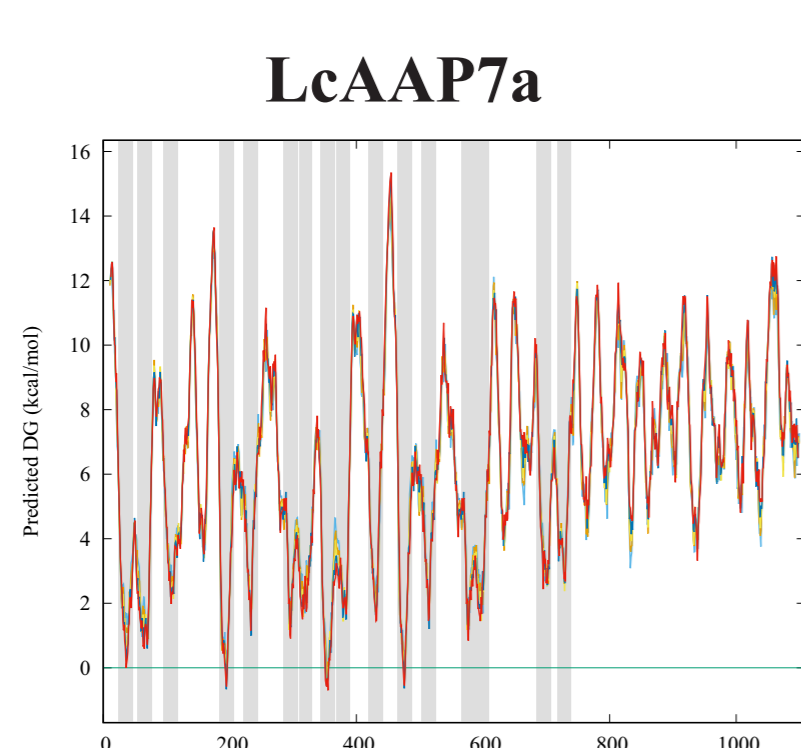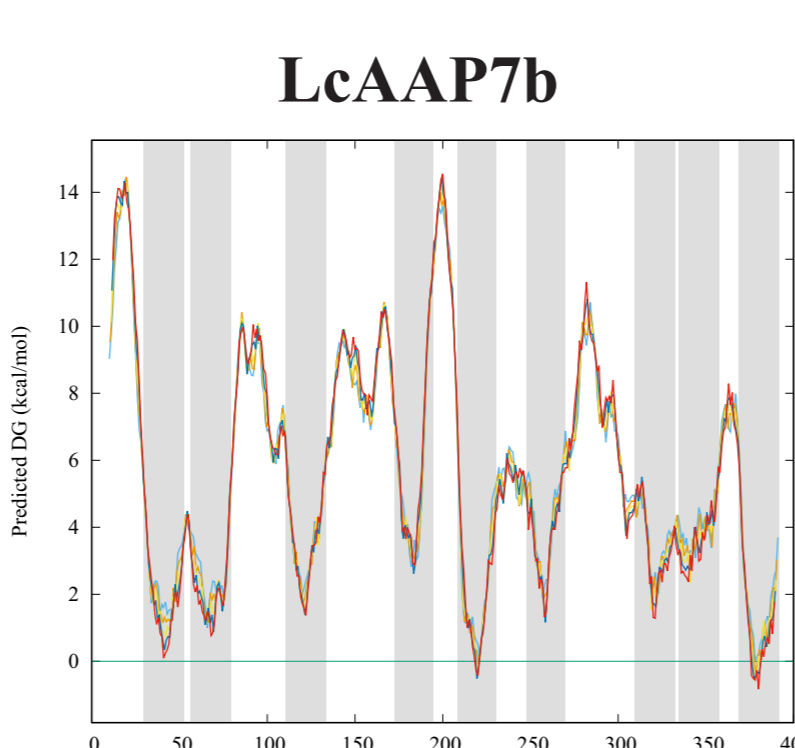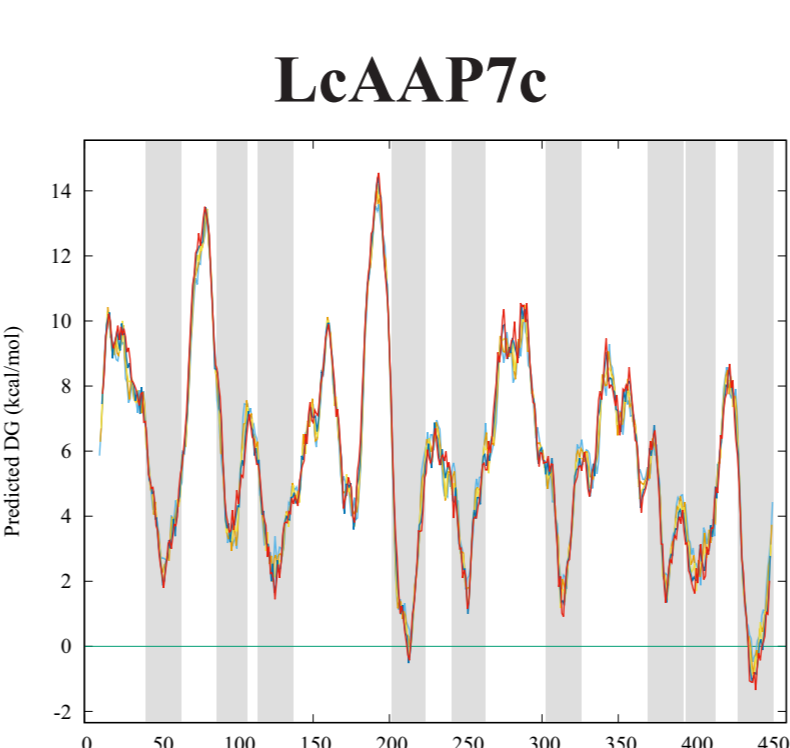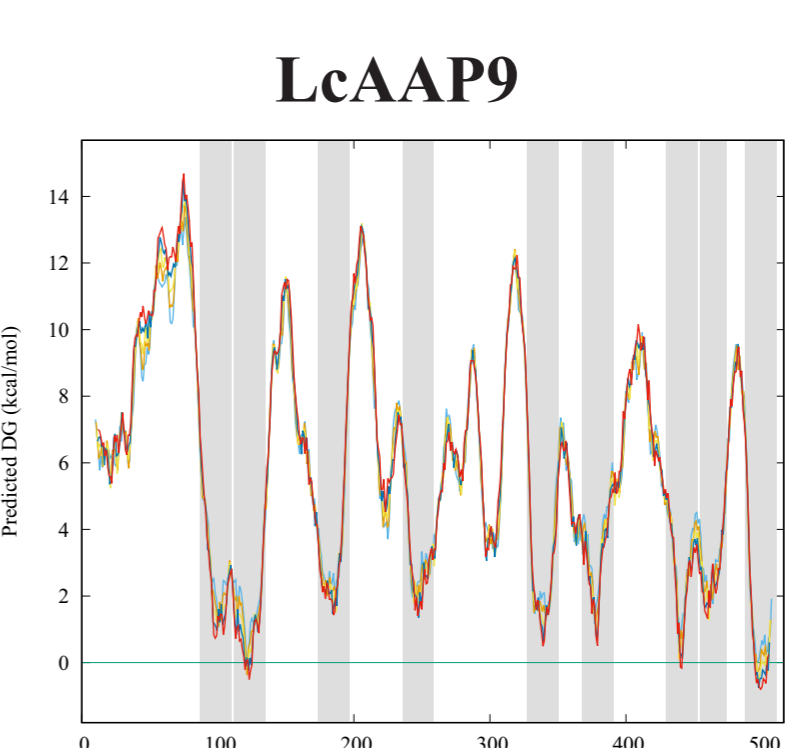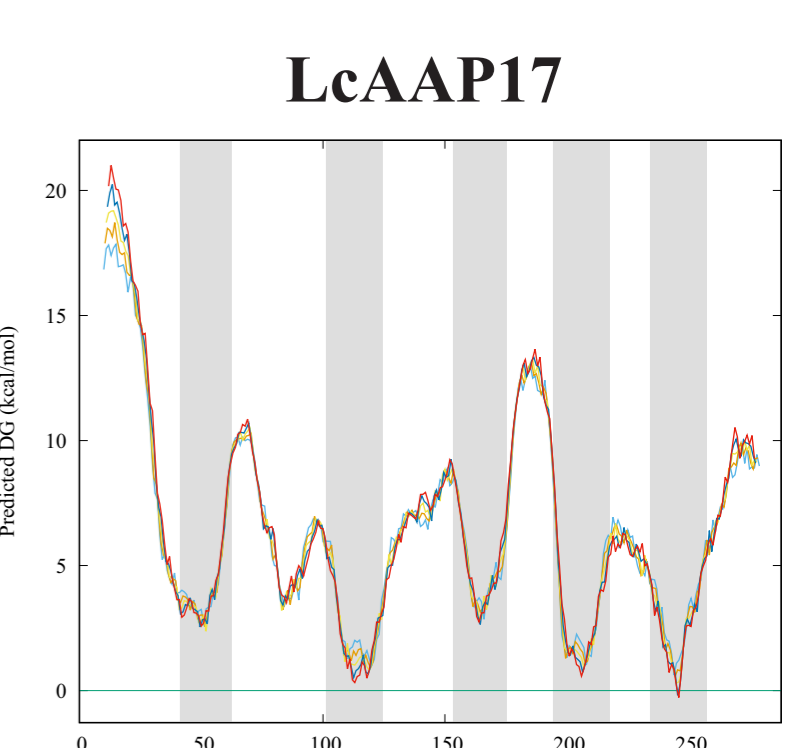

PorT

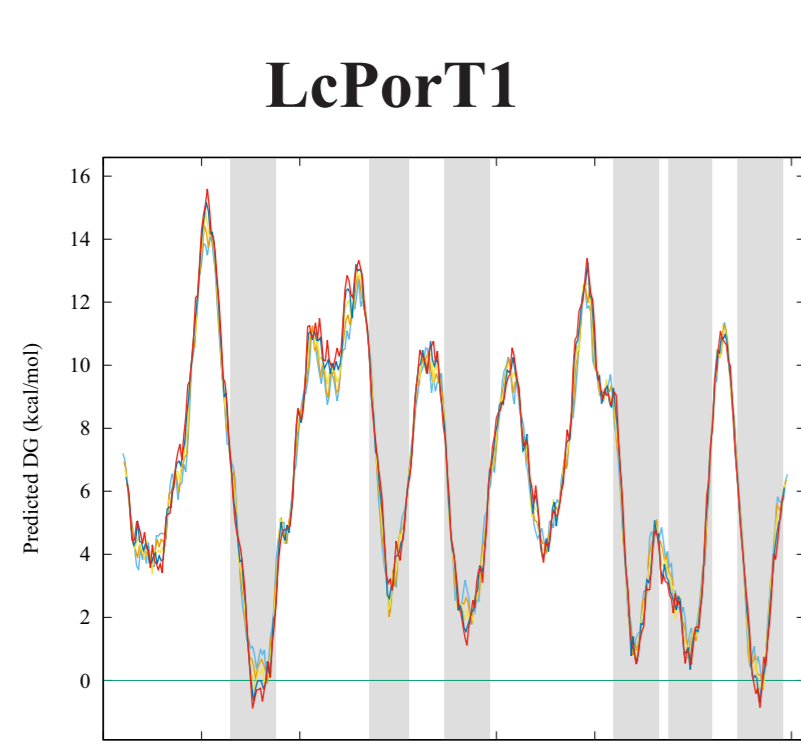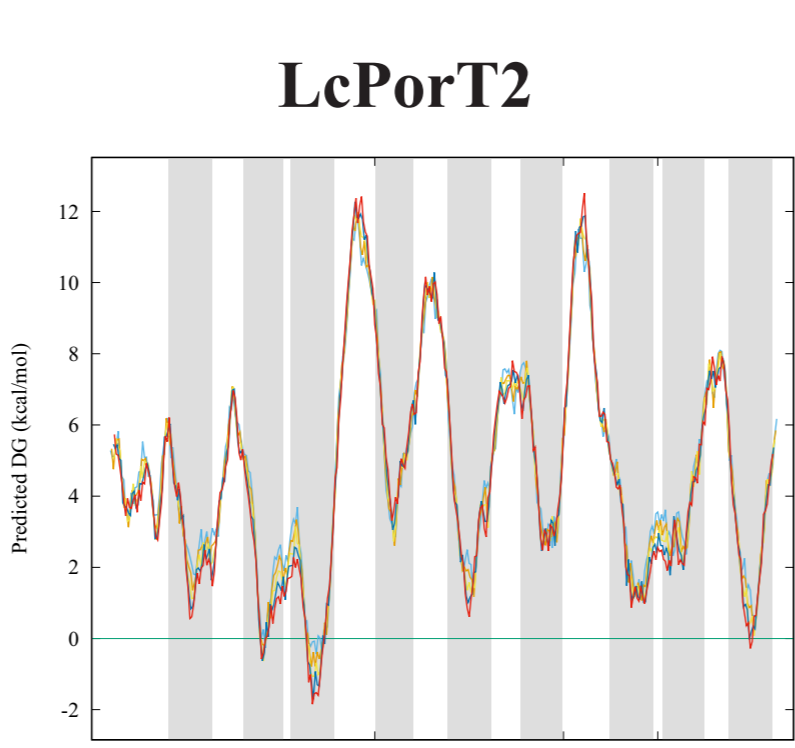

L=19 ———  
L=20 ———  
L=21 ———  
L=22 ———  
L=23 ———

Supplement: Supplementary file 1 [file ijms-23-04765-s001.zip › Figure S3.pdf]
