# Supplementary material for: Identification, Phylogenetic and Expression Analyses of the AAAP Gene Family in Liriodendron chinense Reveal Their Putative Functions in Response to Organ and Multiple Abiotic Stresses
Source: Int J Mol Sci. 2022 Apr 26;23(9):4765. doi: 10.3390/ijms23094765 (PMC9100865; doi:10.3390/ijms23094765)
Supplement: Supplementary file 1 [file Figure S2.pdf › Figure S2.pdf]

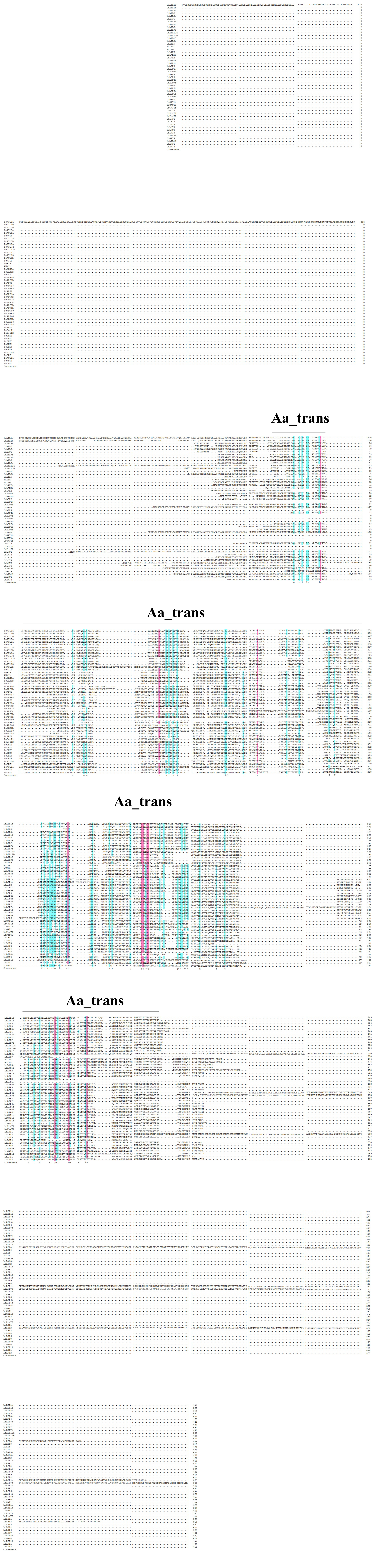
Aa_trans
Aa_trans
Aa_trans
Aa_trans
